# Human-induced pluripotent stem cell-derived microglia integrate into mouse retina and recapitulate features of endogenous microglia

**DOI:** 10.7554/eLife.90695

**Published:** 2024-11-08

**Authors:** Wenxin Ma, Lian Zhao, Biying Xu, Robert N Fariss, T Michael Redmond, Jizhong Zou, Wai T Wong, Wei Li

**Affiliations:** 1 Retinal Neurophysiology Section, National Eye Institute Bethesda United States; 2 Genetic Engineering Core, National Eye Institute Bethesda United States; 3 Immunoregulation Section, National Eye Institute Bethesda United States; 4 Biological Imaging Core, National Eye Institute Bethesda United States; 5 Molecular Mechanisms Section, National Eye Institute Bethesda United States; 6 iPSC Core, National Heart, Lung, and Blood Institute Bethesda United States; 7 Tiresias Bio Half Moon Bay United States; University of Virginia United States; Boston Children's Hospital United States

**Keywords:** human iPSC, microglia, PLX-5622, retinal transplantation, RPE, NaIO_3_, Mouse

## Abstract

Microglia exhibit both maladaptive and adaptive roles in the pathogenesis of neurodegenerative diseases and have emerged as a cellular target for central nervous system (CNS) disorders, including those affecting the retina. Replacing maladaptive microglia, such as those impacted by aging or over-activation, with exogenous microglia that can enable adaptive functions has been proposed as a potential therapeutic strategy for neurodegenerative diseases. To investigate microglia replacement as an approach for retinal diseases, we first employed a protocol to efficiently generate human-induced pluripotent stem cell (hiPSC)-derived microglia in quantities sufficient for in vivo transplantation. These cells demonstrated expression of microglia-enriched genes and showed typical microglial functions such as LPS-induced responses and phagocytosis. We then performed xenotransplantation of these hiPSC-derived microglia into the subretinal space of adult mice whose endogenous retinal microglia have been pharmacologically depleted. Long-term analysis post-transplantation demonstrated that transplanted hiPSC-derived microglia successfully integrated into the neuroretina as ramified cells, occupying positions previously filled by the endogenous microglia and expressed microglia homeostatic markers such as P2ry12 and Tmem119. Furthermore, these cells were found juxtaposed alongside residual endogenous murine microglia for up to 8 months in the retina, indicating their ability to establish a stable homeostatic state in vivo. Following retinal pigment epithelial cell injury, transplanted microglia demonstrated responses typical of endogenous microglia, including migration, proliferation, and phagocytosis. Our findings indicate the feasibility of microglial transplantation and integration in the retina and suggest that modulating microglia through replacement may be a therapeutic strategy for treating neurodegenerative retinal diseases.

## Introduction

Microglia are the innate immune cells of the central nervous system (CNS), including the retina, and play pivotal roles in neuronal (Puñal et al., 2019; Anderson et al., 2019; Huang et al., 2012) and vascular development (Checchin et al., 2006; Ritter et al., 2006; Kubota et al., 2009), normal synapse formation (Stevens et al., 2007; Schafer et al., 2012; Schafer and Stevens, 2015; Hong et al., 2016), maintaining local homeostasis in the neural environment (Li and Barres, 2018; Colonna and Butovsky, 2017; Sierra et al., 2013), and the regulation of immune activity (Okunuki et al., 2019). Conversely, they are also implicated in driving pathologic progression in various retinal diseases, including age-related macular degeneration (AMD) (Combadière et al., 2007; Ma et al., 2009, Ma et al., 2012; Karlstetter et al., 2015), glaucoma (Bosco et al., 2019; Ramírez et al., 2020), diabetic retinopathy (Xu and Chen, 2017), and uveitis (Broderick et al., 2002; Okunuki et al., 2019; Zhou et al., 2020).

Under homeostatic conditions in the adult retina, microglial cells are predominantly distributed in the inner plexiform layer (IPL) and outer plexiform layer (OPL) and vigilantly survey environmental changes through dynamic surveying behavior in their ramified processes (Lee et al., 2008). Their presence and homeostatic function are crucial for maintaining normal retinal functions, including the maintenance of synaptic function and integrity (Wang et al., 2016). Under normal conditions, microglial cells sustain equilibrium in their endogenous numbers via slow self-renewal (Réu et al., 2017). However, with the onset of pathology, this homeostasis can be disrupted following microglia activation, migration, and proliferation. Microglia repopulation in the retina following perturbation is achieved through both the proliferation of endogenous microglia and the infiltration of peripheral monocytes (Ma et al., 2017; Huang et al., 2018; Zhang et al., 2018).

Studies of microglia cell repopulation have indicated that retinal resident microglia can sustain their population with the local microglial cell dividing and migration if any perturbations do not exceed the threshold of the recovery speed by local neighbor microglia. However, in cases of more severe retinal injury or infection that cause significant redistribution of endogenous microglia, the reestablishment of retinal microglial homeostasis will, in addition, involve peripheral monocytes that infiltrate into the retina to take up residence as macrophages. The ability of the retina to incorporate exogenous monocytic cells suggests that microglia cell replacement employing exogenously introduced microglia may be feasible and can exert therapeutic effects post-injury. Inhibiting retinal microglia over-activation has shown efficacy in animal models of retinal injury (Zhao et al., 2011; Karlstetter et al., 2015; Au and Ma, 2022) and potential signal in early-phase clinical trials (Cukras et al., 2012). These observations suggest that the depletion of maladaptive microglia in pathological contexts and their replacement with microglia that have a more homeostatic phenotype may constitute a potential therapeutic strategy.

Studies of microglia have largely been performed in rodent-derived models, largely due to the accessibility of various transgenic disease models. However, several studies have indicated that genetic and functional differences exist between murine and human microglia (Dawson et al., 2018; Friedman et al., 2018; Ueda et al., 2016). For instance, microglia-expressed genes CD33 and CR1, which have been associated with Alzheimer’s disease (AD) risk in genome-wide association studies, lack reliable orthologs in mice (Hasselmann and Blurton-Jones, 2020). Additionally, over half of the AD risk genes that are enriched in microglia demonstrate <70% sequence homology between humans and mice (Hasselmann and Blurton-Jones, 2020). There are also significant differences in the levels of protein expression of some microglia-expressed complement factors and inflammatory cytokines between humans and mice (Galatro et al., 2017; Gosselin et al., 2017; Smith and Dragunow, 2014). As a result, microglia from murine models may not accurately represent those found in human conditions (Burns et al., 2015), limiting their translational potential.

To study microglia of human origin, some investigators have attempted to isolate microglial cells from human tissue. However, owing to limitations in sample availability, and the rapid transcriptomic changes that ex vivo microglia undergo post-isolation, these studies have been technically constrained (Bohlen et al., 2017; Butovsky et al., 2014; Gosselin et al., 2017). As an alternative to primary microglia, human-induced pluripotent stem cells (iPSCs) offer a wealth of possibilities and have been increasingly used to generate human microglia via differentiation in vitro (Muffat et al., 2016; Pandya et al., 2017; Abud et al., 2017; Douvaras et al., 2017; Haenseler et al., 2017; Takata et al., 2017). This approach has enabled the generation of a large quantity of cells of a specified genetic background, enabling the creation of in vivo human microglia cell models through xenotransplantation of human-induced pluripotent stem cell (hiPSC)-derived microglia into the murine CNS (Abud et al., 2017; Svoboda et al., 2019; Parajuli et al., 2021; Xu et al., 2020a; Chadarevian et al., 2023).

In this study, we adopted a previously published protocol appropriate for culturing hiPSC-derived microglial cells (Muffat et al., 2016; Pandya et al., 2017; Abud et al., 2017; Douvaras et al., 2017; Haenseler et al., 2017; Takata et al., 2017). We characterized microglia differentiation by examining the RNA and protein expression levels of microglia-enriched genes. We also assessed the inflammatory responses and phagocytic functions of hiPSC-derived microglia in vitro. We then established a human iPSC-derived microglia cell model through the xenotransplantation of human iPSC-derived microglial cells into the retina of an adult mouse. We found that when transplanted by subretinal injection, human iPSC-derived microglial cells were able to migrate into the retina where native retinal microglia reside and acquire a morphology resembling endogenous mouse microglia, and express microglia signature markers. These grafted cells persisted in the mouse retina for at least 8 months and responded to retinal pigment epithelial (RPE) cell injury in ways resembling endogenous mouse microglia. Xenografting of hiPSC-derived microglia into mouse retina has the promise of being used to create in vivo models of retinal disease and injury to evaluate the preclinical efficacy of potential therapeutic agents, as well as to evaluate microglia transplantation itself as a potential therapeutic intervention.

## Results

### Differentiation and characterization of human iPSC-derived microglia

We used five distinct human iPSC lines for microglia cell differentiation, including the first-available iPSC line, KYOUDXR0109B, from ATCC; and four other lines NCRM6, MS19-ES-H, ND2-AAVS1-iCAG-tdTomato, and NCRM5-AAVS1-CAG-EGFP, from the National Heart, Lung, and Blood Institute (NHLBI). Our approach to microglia differentiation was informed by our previous work with primary mouse retinal microglia cell culture (Ma et al., 2009) and a variety of established microglia cell differentiation protocols (Muffat et al., 2016; Pandya et al., 2017; Abud et al., 2017; Douvaras et al., 2017; Haenseler et al., 2017; Takata et al., 2017). We opted for the myeloid progenitor/microglia cell floating culture method (van Wilgenburg et al., 2013; Haenseler et al., 2017) for its simplicity, efficiency, and consistency, which enables the generation of a large and uniform population of microglial cells.

**Figure 1.**
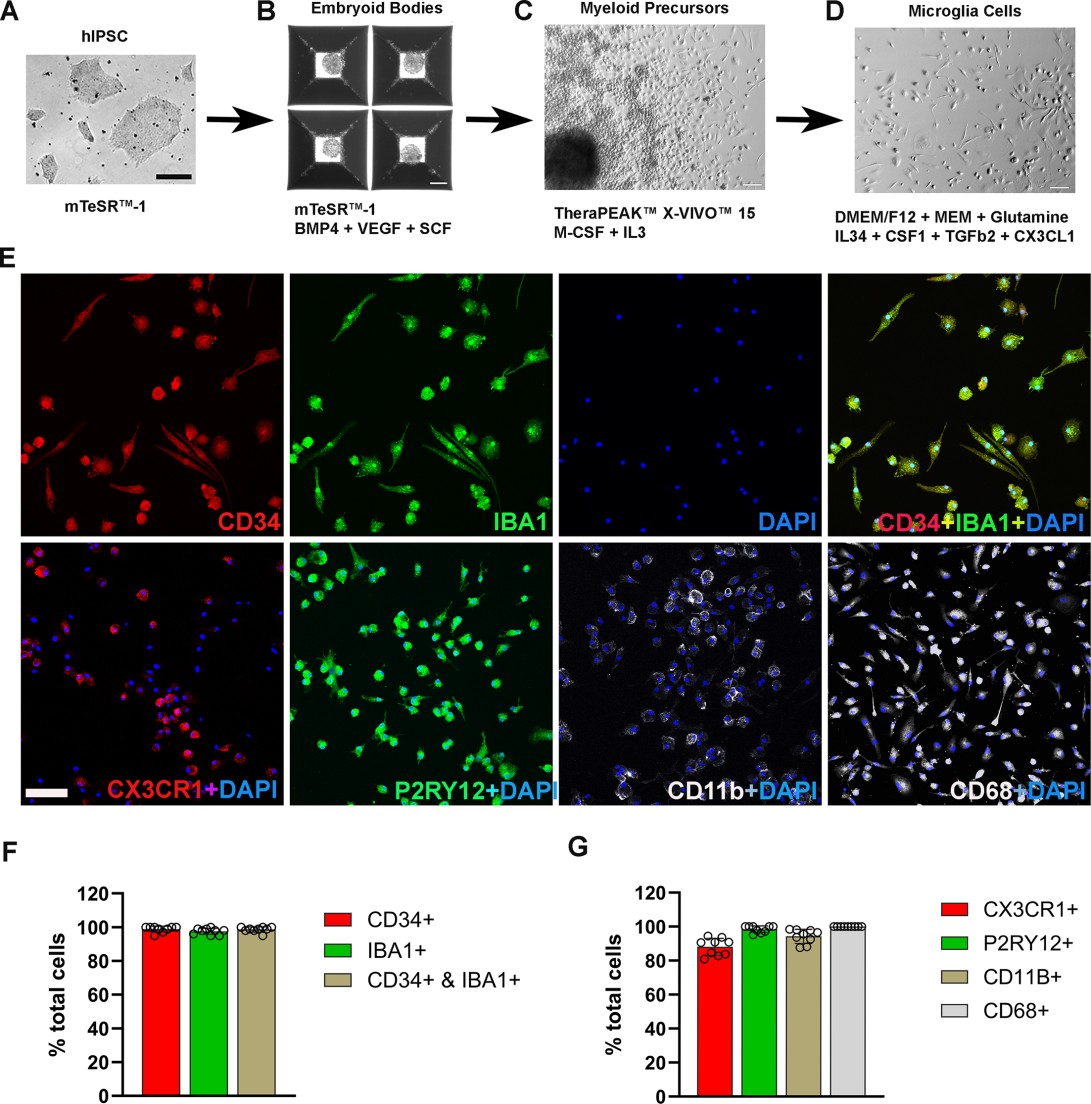
Differentiation and characterization of human-induced pluripotent stem cell (iPSC)-derived microglia. (**A**) Human iPSCs were cultured in a 6-well plate. Scale bar = 200 µm. (**B**) Embryoid body formation was enabled in AggreWell800 plate at day 8 in culture medium mTeSR1 plus BMP4, VEGF, and SCF. Scale bar = 200 µm. (**C**) Image of a myeloid precursor cluster following 1 month culture of embryoid bodies in TheraPEAK X-vivo-15 Serum-free Hematopoietic Cell Medium with added M-CSF and IL3. Scale bar = 50 µm. (**D**) Image of microglial cells in maturation culture for 2 weeks with Dulbecco's Modified Eagle Medium (DMEM)/F12 plus non-essential amino acids, glutamine, IL34, CSF1, TGFb2, and CX3CL1. Scale bar = 50 µm. (**E**) Immunohistochemical staining for *Iba1* and human CD34, CX3CR1, P2RY12, CD11b, and CD68. Scale bar = 100 µm. (**F**) Cell counts and colocalization analysis of (**F**) CD34- and Iba1-positive cells and (**G**) positivity for myeloid cell markers CX3CR1, CD11b, activation marker CD68, and microglia marker P2RY12 in differentiated microglia.

The differentiation process involved three key stages: embryoid body (EB) formation, myeloid progenitor cell generation, and microglia cell maturation (Figure 1A–D). Following myeloid differentiation, floating myeloid progenitor cells were harvested and allowed to differentiate further for 2 weeks in 6-well plates/flask under conditions promoting microglial differentiation. We modified this step to include additional differentiation factors in the differentiation medium, including IL34, CSF1, CX3CL1, TGFB1, and TGFB2. We found that these factors promoted microglial morphological ramification and process elongation. Immunohistochemical analysis of the resulting cells showed that among CD34(+) cells, 98.6% were immunopositive for IBA1 (Figure 1E, F), and 98.5% were immunopositive for P2RY12 (Figure 1E, G). Immunostaining with myeloid cell markers CX3CR1, CD68, and CD11b showed positivity in 88%, 99.7%, and 94.3%, respectively, demonstrating the high efficiency of differentiation achieved by this procedure (Figure 1—figure supplement 1). Most of the resulting hiPSC-derived microglia showed spindle-shaped morphologies, with some displaying short ramifications in their processes (Figure 1E), resembling those observed in primary mouse retinal microglia cultures (Ma et al., 2009). Floating myeloid progenitor cell harvest could be performed repeatedly over 3 months following culture establishment, providing a steady and consistent supply for further microglia differentiation and generation.

**Figure 1—figure supplement 1.**
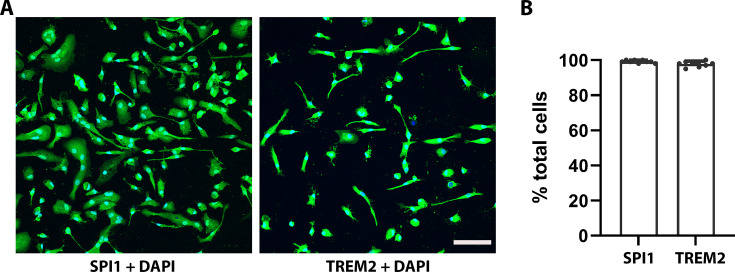
Immunocytochemistry staining with human SPI1 and TREM2. (**A**) Confocal images showed SPI1 and TREM2 staining. Scale bar = 100 μm. (**B**) SPI1- and TREM2-positive cells are 99.7% and 99.4%, respectively, in entire DAPI+ cell counts.

**Figure 2.**
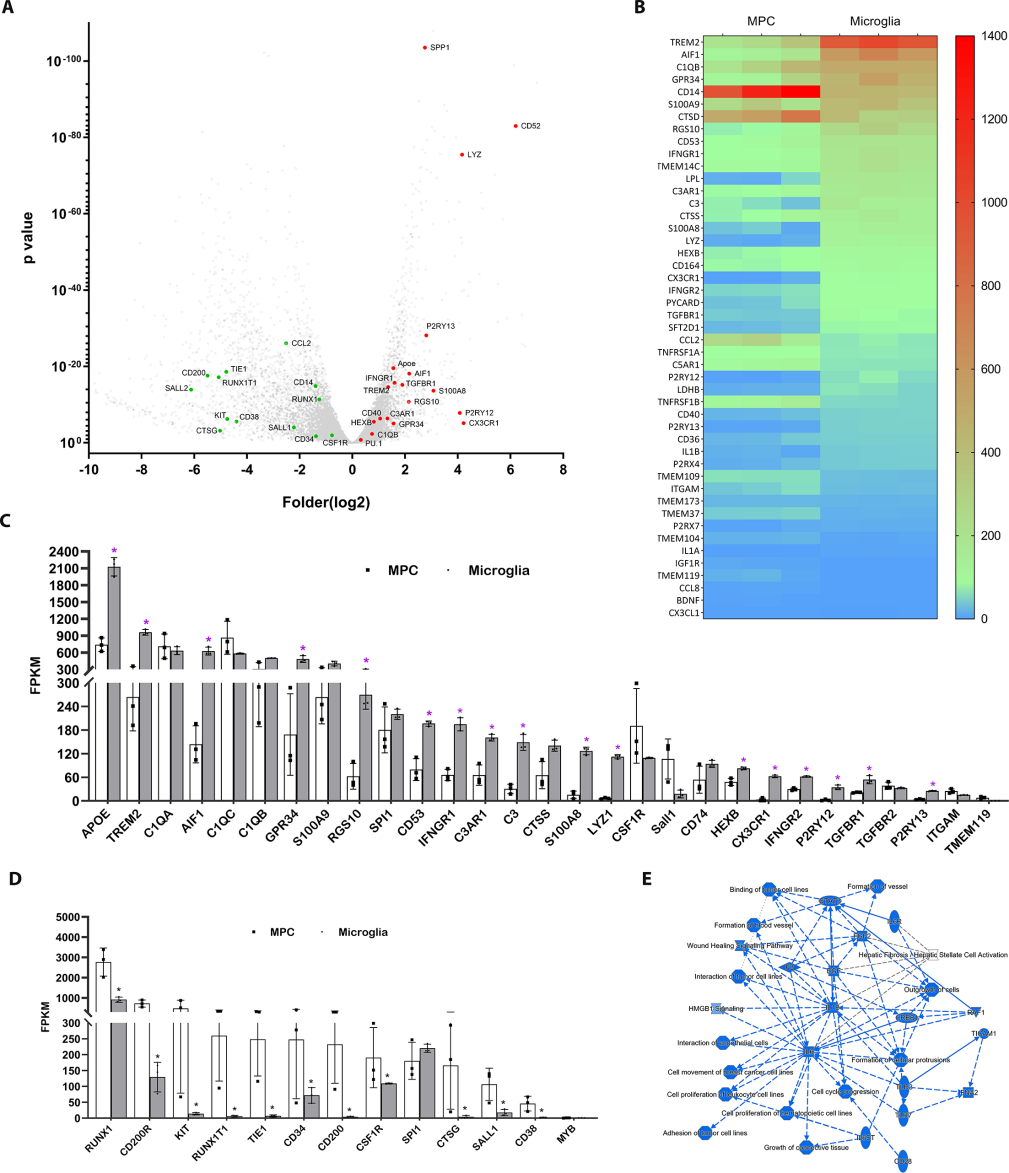
Profiling of genes differentially expression between differentiated microglial cells vs. myeloid progenitor cells (MPCs) using bulk RNAseq analysis. (**A**) Volcano plot showing representative genes that were either upregulated (red) or downregulated (green) in differentiated microglia vs. MPCs. (**B**) Heat map showing increased expression of microglia-enriched genes in differentiated microglia (Supplementary file 1). (**C**) Histogram comparing the expression levels of microglia-enriched genes in terms of Fragments Per Kilobase of transcript per Million mapped reads (FPKM). *p < 0.05. (**D**) Histogram comparing expression levels of myeloid cell lineage genes in human-induced pluripotent stem cell (iPSC)-derived MPC and microglia cells using FPKM. *p < 0.05. (**E**) Graphic signaling pathway analysis with Ingenuity Pathway Analysis (IPA) highlighting *IL6* and *IL1B* as signaling hubs in differential gene expression patterns (Supplementary file 1) in differentiated microglia vs. MPCs.

A comparative RNAseq analysis between differentiated hiPSC-derived microglia collected at the end of the protocol vs. floating myeloid progenitor cells revealed a significant upregulation of microglia-enriched gene expression following microglial differentiation, including *Cx3cr1*, *P2ry12*, *P2ry13*, *Aif1*, *Trem2*, *Gpr34*, *CD53*, *CTSS*, and *C3aR1* (Figure 2A–C). Moreover, hiPSC-derived microglia exhibited higher expression of genes associated with inflammation, apoptosis regulation, phagocytosis, lipid metabolism, and immune responses. The floating myeloid progenitor cells showed higher expression of hematopoietic/myeloid cell lineage genes (Figure 2D). Ingenuity Pathway Analysis (IPA) of differentially expressed genes identified IL6, IL1B, and STAT3 as central signaling hubs critical to the regulation of inflammatory responses in microglia (Figure 2E, Figure 2—figure supplement 1, Supplementary file 1). We also compared the gene expression profiles between differentiated hiPSC-derived microglial cells vs. human microglia isolated ex vivo from the fetal and adult brain (Figure 2—figure supplements 2–4 Supplementary files 2 and 3; Abud et al., 2017; Douvaras et al., 2017; Muffat et al., 2016; Böttcher et al., 2019; van der Poel et al., 2019; Eisenberg and Levanon, 2013). The results of the correlation analysis indicated that hiPSC-derived microglial cells demonstrated an expression profile comparable to those in fetal and adult microglia in vivo but were more distinct from those in monocytes and inflammatory monocytes. Thus, our method of obtaining differentiated microglia can reliably and efficiently generate a large population of homogenous functional microglial cells of human origin.

**Figure 2—figure supplement 1.**
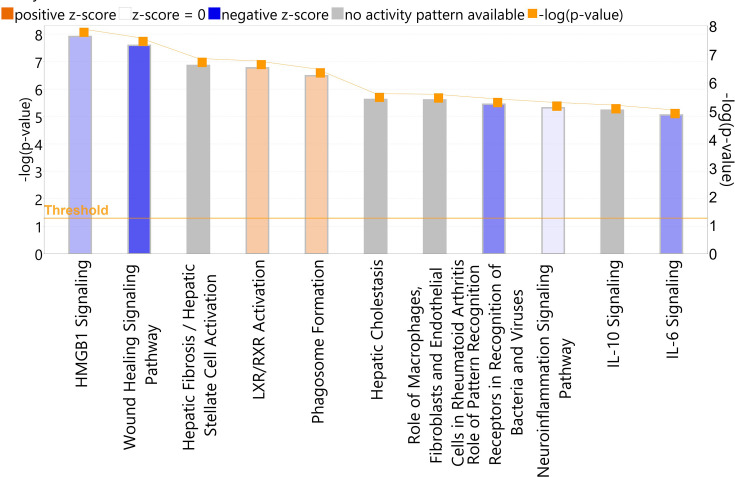
Analysis results of the canonical pathway of complete differentiated human-induced pluripotent stem cell (hiPSC)-derived microglia vs. myeloid progenitor cells with Ingenuity Pathway Analysis (IPA) with the enriched microglia genes (Supplementary file 1).

**Figure 2—figure supplement 2.**
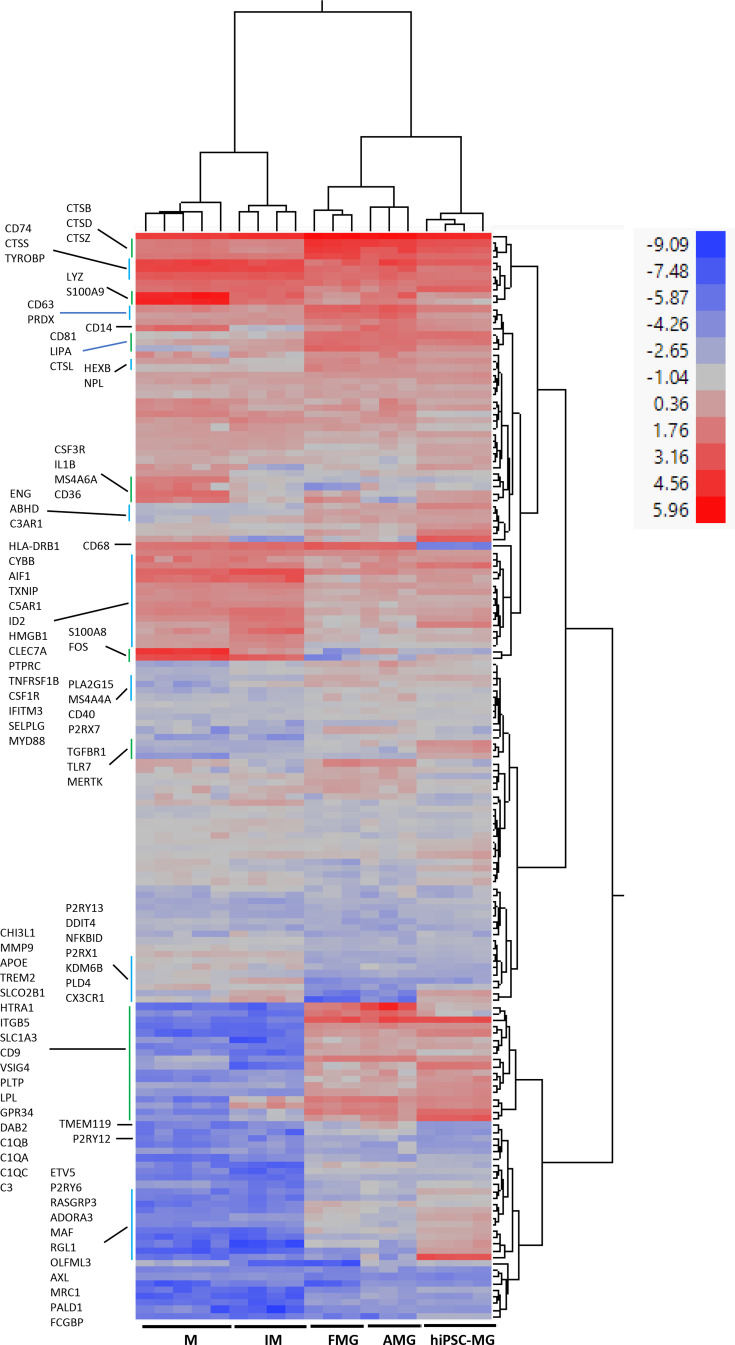
Hierarchical cluster analysis on microglia-enriched genes among human-induced pluripotent stem cell (hiPSC)-derived microglial cells (hiPSC-MG) and human adult brain microglia cells (AMG), fetal brain microglia cells (FMG), inflammatory monocytes (IM), monocytes (M). The human microglia gene panel combined our mouse microglia-enriched genes and human microglia-enriched genes (Abud et al., 2017; Muffat et al., 2016; Douvaras et al., 2017; Böttcher et al., 2019; van der Poel et al., 2019). The total microglia-enriched gene list contains 203 genes (Supplementary file 2), which used to be extracted from the gene profile of human-induced pluripotent stem cell (hiPSC)-MG and downloaded human adult microglia (AMG), fetal microglia (FMG), inflammatory monocyte (IM) and monocytes (M) (GSE 178846, Abud et al., 2017). 188 genes (Supplementary file 2) were obtained from both gene lists. All the gene counts were normalized with four human cells housekeeping genes C1orf43, RAB7A, REEP5, and VCP (Eisenberg and Levanon, 2013). The hierarchical cluster was analyzed by JMP (JMP Statistical Discovery LLC). Results showed that hiPSC-MG is more comparable with AMG and FMG.

**Figure 2—figure supplement 3.**
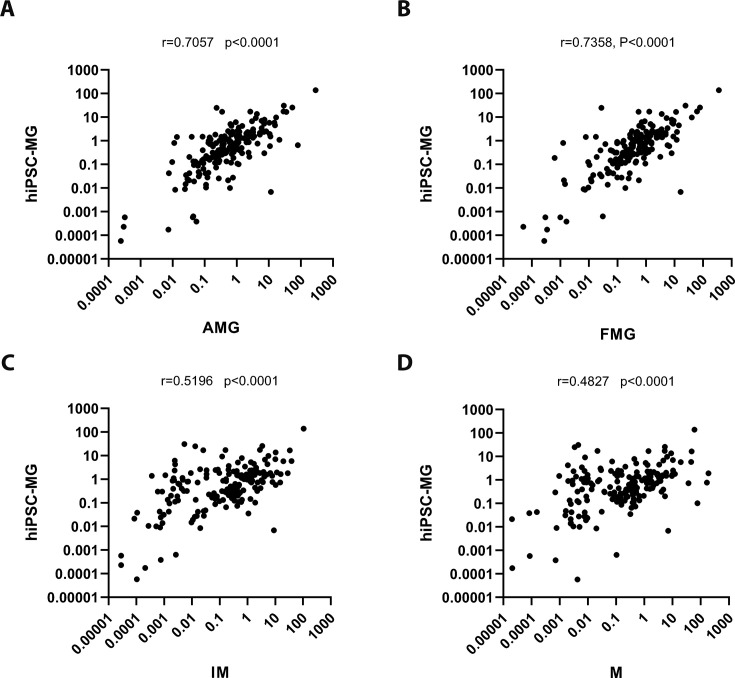
Correlation analysis between the expression levels of microglia-enriched genes in human-induced pluripotent stem cell (hiPSC)-derived microglia cells (hiPSC-MG) vs. those in other myeloid cells. The correlation analysis of 188 human microglia-enriched genes (Supplementary file 2) among human-induced pluripotent stem cell (hiPSC)-derived microglia cells (hiPSC-MG), human adult brain microglia (AMG) cells (**A**), fetal brain microglia (FMG) cells (**B**), inflammatory monocytes (IM) (**C**), and monocytes (M) (**D**) (GSE 178846, Abud et al., 2017), respectively. The images and the analysis results (Prism, GraphPad) showed that expression levels of microglia-enriched genes in hiPSC-MG are more correlated between those in FMG (*r* = 0.7358, p < 0.0001) and AMG (*r* = 0.7057, p < 0.0001).

**Figure 2—figure supplement 4.**
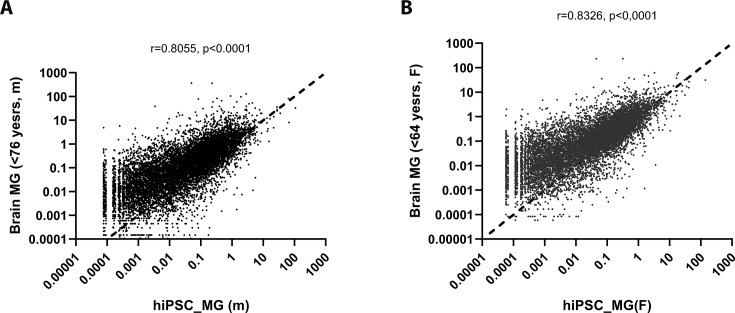
Correlation analysis between the expression levels of genes in human-induced pluripotent stem cell (hiPSC)-derived microglia cells (hiPSC-MG) vs. human donor brain microglia by sex. Correlation comparison of entire gene profiles between male (**A**)/female (**B**) human-induced pluripotent stem cell (hiPSC)-derived microglial cells and male (**A**)/female (**B**) human brain microglia cells (GSE 111972, van der Poel et al., 2019; Supplementary file 3), respectively. The results showed they are significantly correlative (*r* = 0.8055 (M), *r* = 0.8326 (F), p < 0.0001).

### Human iPSC-derived microglia show inflammation responses and phagocytosis activity

**Figure 3.**
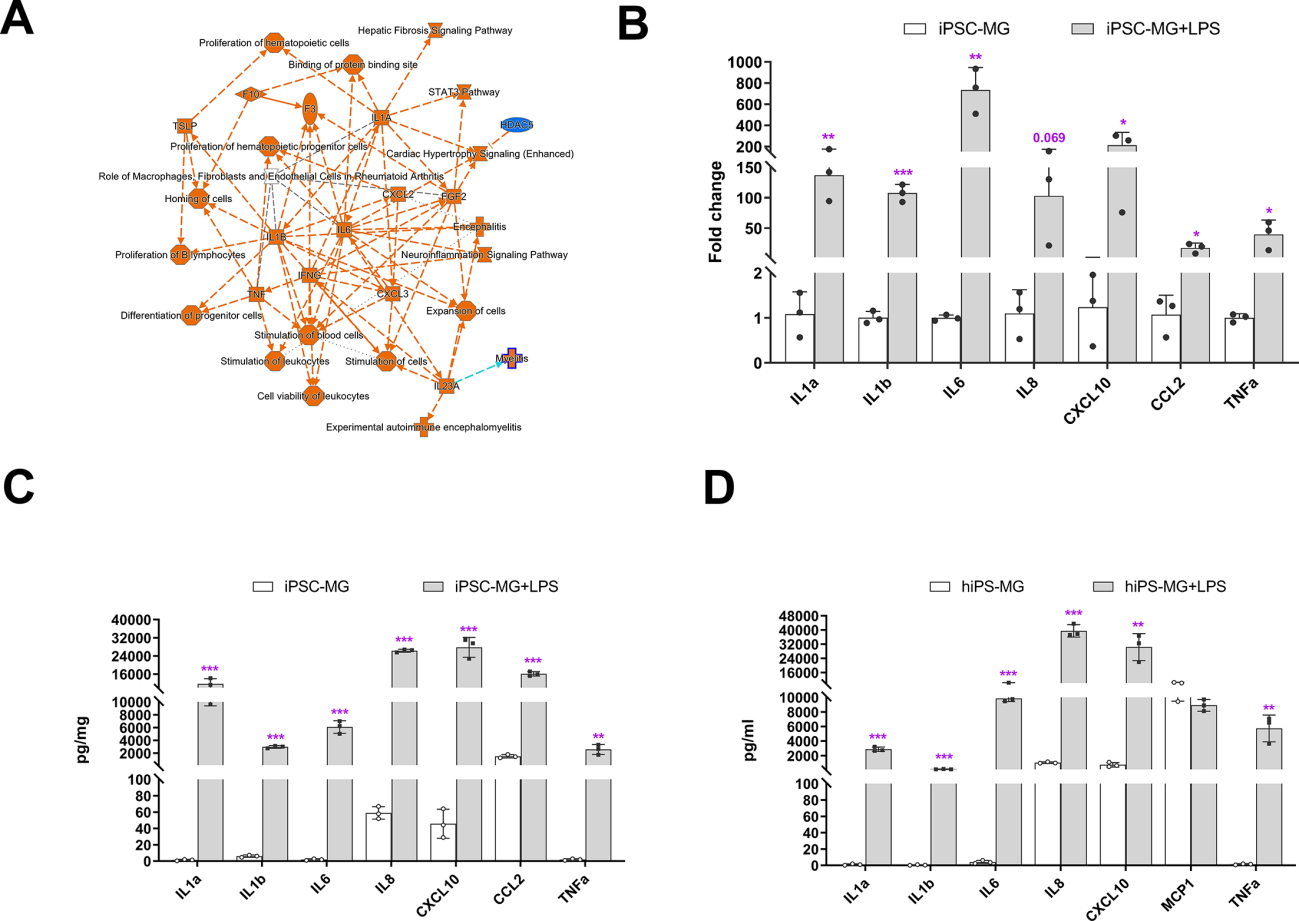
Inflammation responses of human-induced pluripotent stem cell (hiPSC)-derived microglial cells following lipopolysaccharide (LPS) stimulation. (**A**) Ingenuity Pathway Analysis (IPA) showed different gene expression (fold change >twofold, p < 0.05) between LPS-treated and control hiPSC-derived microglial cells, demonstrating activation of core pathways involving *IL6*, *IL1A*, *IL1B*, and *IFNG*. (**B**) Assessment of mRNA expression of selected genes for inflammatory cytokines using quantitative reverse transcription-PCR (qRT-PCR; Oligonucleotide primers are provided in Supplementary 4) demonstrated increased expression following LPS (0.1 µg/ml) stimulation for 6 hr (3-6 replicates). These changes corresponded to increases in the protein expression levels of inflammatory cytokines following 24 hr of LPS stimulation as measured with a Multiplex kit (Millipore) in cell lysate (**C**) and conditioned media (**D**). The data in (**C**) and (**D**) are presented as means ± SEM (3-6 replicates). *p < 0.05, **p < 0.01, ***p < 0.001.

Microglia play crucial roles in mediating inflammatory responses to stimuli and in phagocytosing pathogens. To investigate these functions further, we stimulated hiPSC-derived microglia with lipopolysaccharide (LPS) and analyzed their responses. Transcriptomic profiling using bulk RNAseq revealed that the primary responses to LPS stimulation involved changes in *IL6*, *IL1B*, *IL1A*, *TNFA*, and *IFNG* signaling (Figure 3A, Supplementary file 4), indicating the ability of hiPSC-derived microglia to demonstrate classical activation. This was confirmed through expression analysis using quantitative reverse transcription-PCR (qRT-PCR) and protein multiplex profiling, which showed a 50- to 800-fold increase in the expression of *IL6*, *IL1A*, *IL1B*, *TNFA*, *IL8*, *CXCL10*, and *CCL2* mRNA after 6 hr of LPS stimulation (Figure 3B). Similarly, we observed a significant increase in protein expression levels of these cytokines in cell lysate and culture medium (Figure 3C, D). We also treated hiPSC-derived microglia with IFNγ and a combination of IFNγ + LPS (Figure 3—figure supplement 1), and the results demonstrated that the combination of IFNγ + LPS promoted *IL1A*, *IL1B*, *TNFA*, *CCL8*, and *CXCL10* expression. These findings indicate that hiPSC-derived microglia, akin to microglia in vivo, exhibited strong responses to LPS and IFNγ stimulation.

**Figure 3—figure supplement 1.**
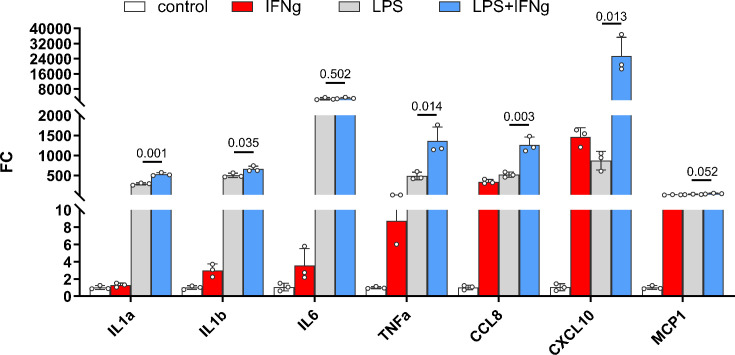
Inflammatory cytokines were produced synthetically by IFNG and LPS in human-induced pluripotent stem cell (hiPSC)-derived microglial cells. hiPSC-derived microglial cells were cultured in 6-well plates for 14 days, 20 ng/ml of human IFNG and 0.5 µg/ml of LPS were added to the wells, respectively, or 20 ng/ml of human IFNG and 0.5 µg/ml of LPS were added to the wells together. After 6 hr of incubation, the cells were washed and harvested into a 1.5-ml Eppendorf tube for RNA isolation with a NucleoSpin RNA/Protein Mini kit (Macherey-Nagel, #740933.50). The cDNA was synthesized with PrimeScript 1st strand cDNA Synthesis Kit (Takara, #6110A). Quantitative reverse transcription-PCR (qRT-PCR) was performed using a SYBR green RT-PCR kit (Affymetrix), using the Bio-Rad CFX96 Touch Real-Time PCR Detection System under the following conditions: denaturation at 95°C for 5 min, followed by 40 cycles of 95°C for 10 s, and then 60°C for 45 s. Threshold cycle (CT) values were calculated and expressed as fold-induction determined using the comparative CT (2ΔΔCT) method. Ribosomal protein S13 (RPS13) and GAPDH were used as internal controls. Oligonucleotide primers are provided in Supplementary 4, the samples have 3 replicates. .

**Figure 4.**
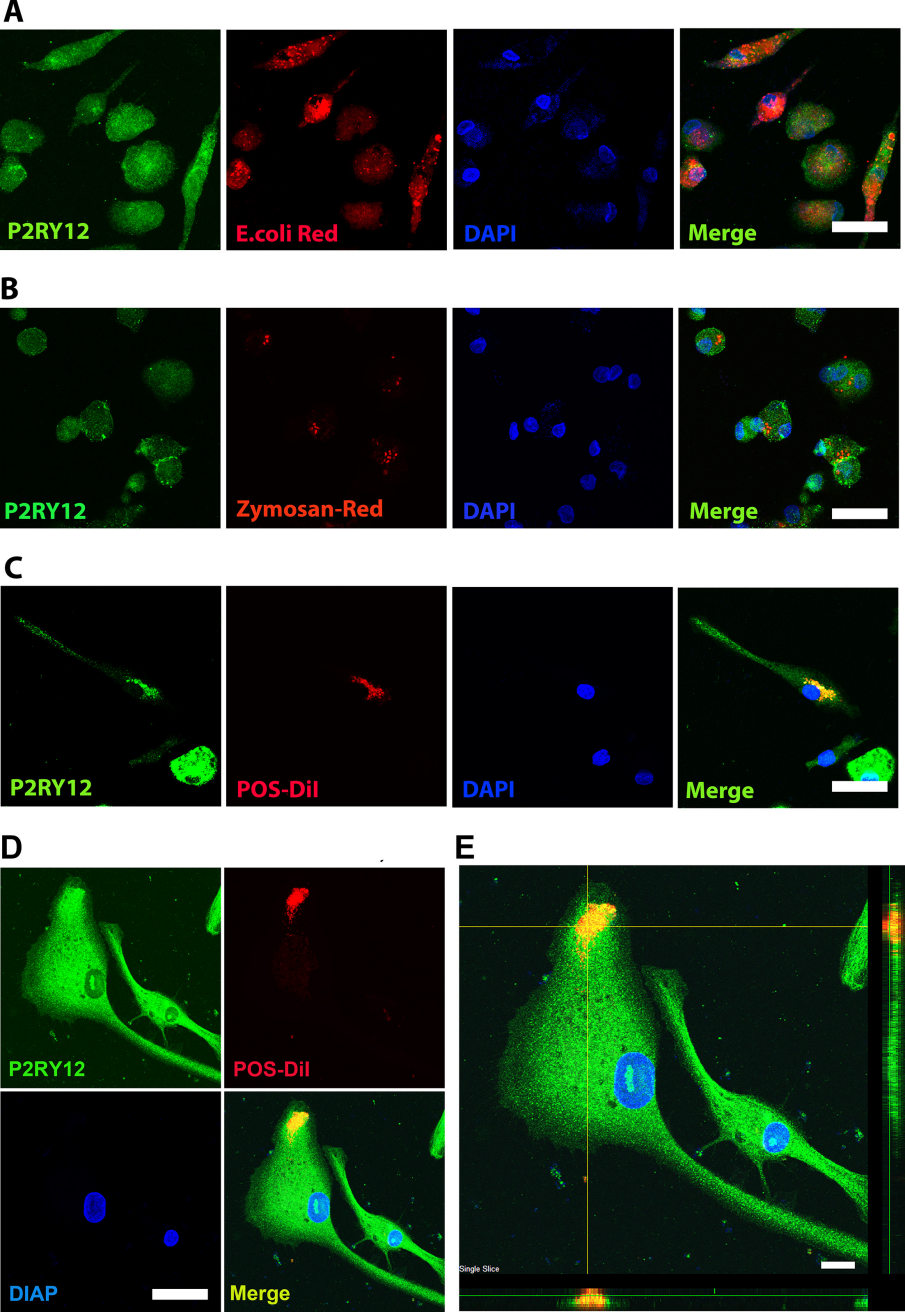
Human-induced pluripotent stem cell (iPSC)-derived microglia demonstrate robust phagocytosis. Human iPSC-derived microglia were incubated for 1 hr in pHrodo Red *E. coli* bioparticles (**A**), pHrodo Red zymosan bioparticles (**B**), DiI-labeled bovine photoreceptor outer segments (POSs) (**C**) and labeled with anti-human *P2RY12* antibody (green) and DAPI. Scale bar = 40 µm. (**D**) A high-magnification view of a POS-containing intracellular vesicle within a labeled microglial cell is shown. Scale bar = 40 µm. (**E**) An overlay of panels in (**D**) with side views. Scale bar = 40µm.

Microglia are local immune cells in the CNS, functioning as phagocytes involved in clearing apoptotic or necrotic cells, and cell debris (Green et al., 2016), remodeling neuronal connectivity by engulfing synapses, axonal and myelin debris (Paolicelli et al., 2011), and removing pathogens by direct phagocytosis (Nau et al., 2014). To assess the phagocytic capability of hiPSC-derived microglia, we exposed them to three different types of bioparticles: *Escherichia coli* bacteria, zymosan, and bovine photoreceptor outer segments (POSs) (Figure 4A–D). The cells altered their morphology in response and rapidly internalized the fluorescent-labeled particles (Figure 4A–D, Figure 4—figure supplements 1 and 2). The engulfed bioparticles were condensed into perinuclear aggregates, likely within lysosomal bodies. They also demonstrated concurrent morphological changes into amoeboid-shaped cells (Figure 4D, E), resembling phenotypes demonstrated by retinal microglia phagocytosing photoreceptors in the context of photoreceptor degenerative pathologies in vivo (Zhao et al., 2015).

**Figure 4—figure supplement 1.**
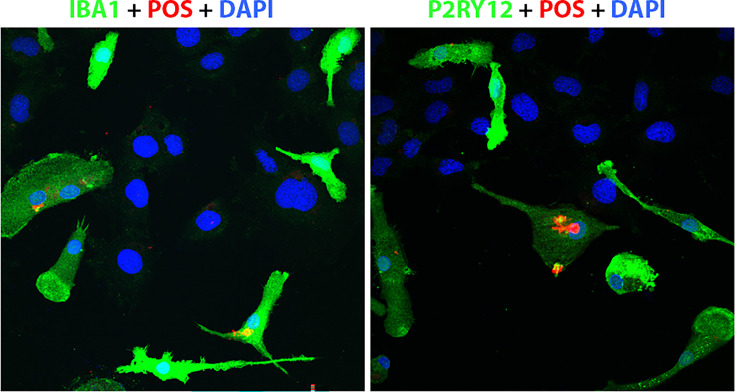
Floating myeloid progenitor cells were cultured in a 2-well slide chamber overnight, and then the DiI-labeled bovine photoreceptor outer segments were added to the chamber and incubated for 1 hr. The cells were fixed with 4% paraformaldehyde (PFA) for 20 min and stained with IBA1 and hP2RY12, respectively. The DiI-labeled bovine photoreceptor outer segments can only be engulfed by IBA1- or P2RY12-positive microglia cells. Scale bar = 16 µm.

**Figure 4—figure supplement 2.**
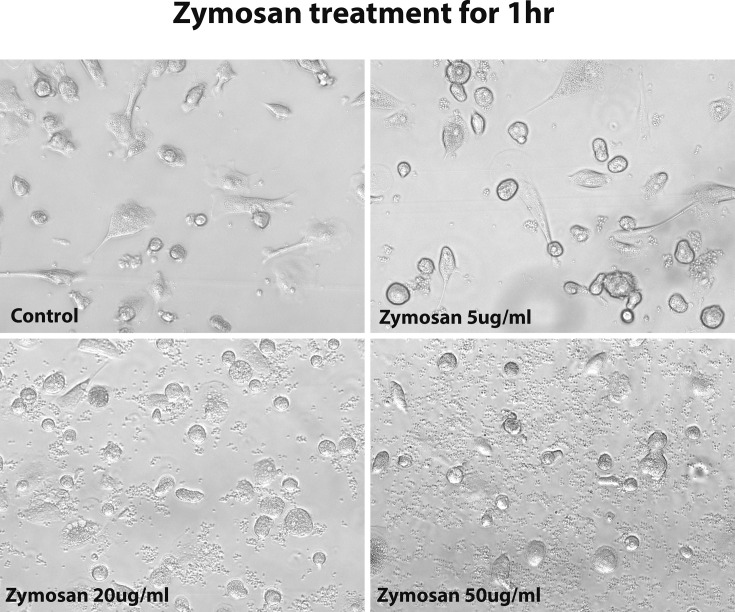
The morphology of human-induced pluripotent stem cell (hiPSC)-derived microglia cells under 1 hr zymosan treatment with different concentrations. After 1 hr of treatment with zymosan, 5 µg/ml concentration did not change the microglia cell morphology, but over 20 µg/ml concentration changed the microglia morphology to an ameboid round shape.

### Transition of hiPSC-derived microglial cells to a homeostatic state within the mouse retina following xenotransplantation in vivo

**Figure 5.**
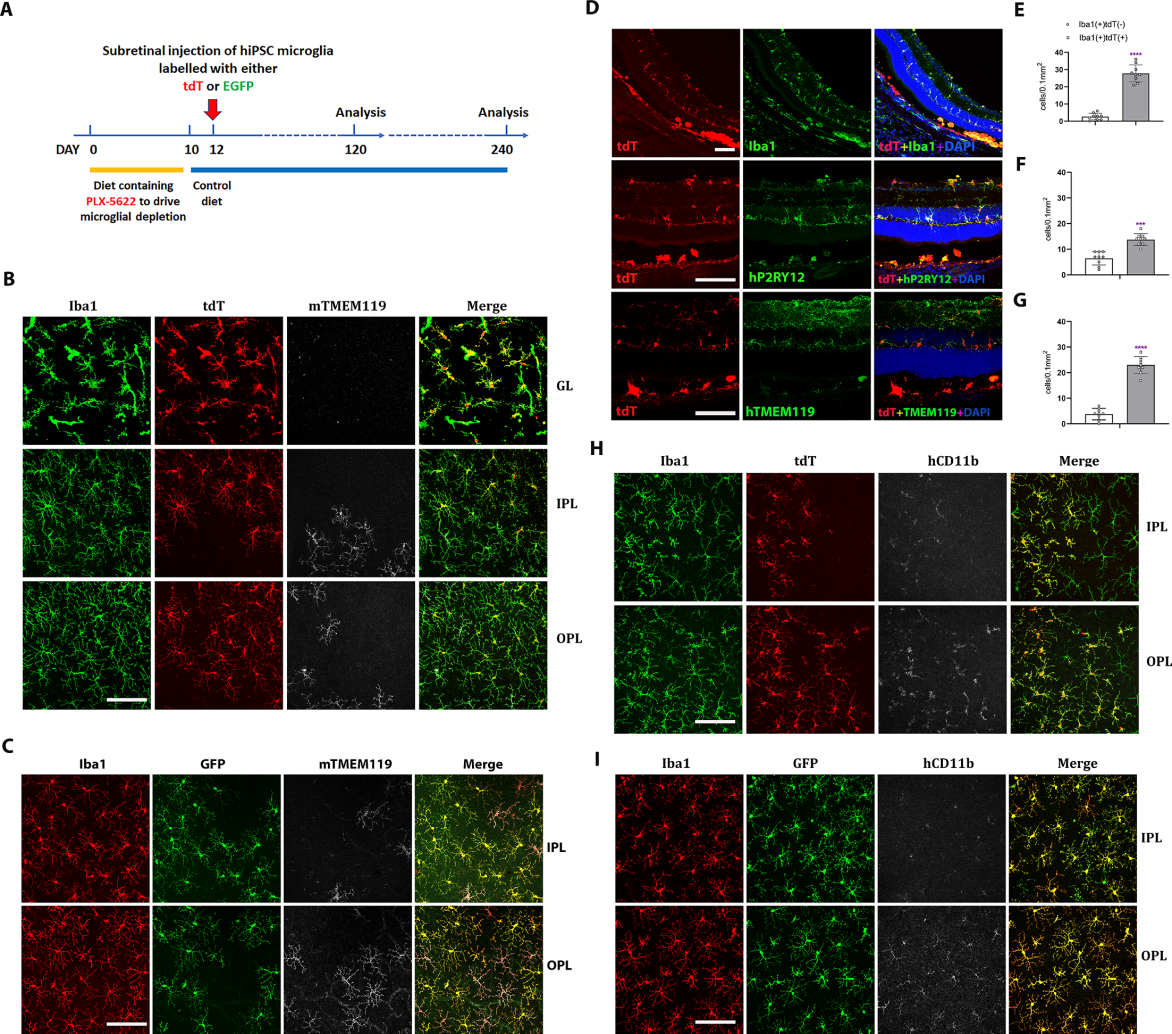
Xenotransplanted human-induced pluripotent stem cell (iPSC)-derived microglial cells into recipient mouse retina in vivo demonstrate recapitulation of endogenous distribution, cellular morphology, and stable integration for up to 4 months. (**A**) The schematic diagram shows the timeline for transplantation experiments. Two-month-old adult transgenic Rag2^−/−^;IL2rg^−/−^;hCSF1^+/+^ mice were fed a PLX-5622-containing diet for 10 days before switching to standard chow. Two days following the resumption of standard chow, human iPSC-derived microglial cells expressing either tdTomato or EGFP were xenotransplanted into the subretinal space via subretinal injection (5000 cells in 1 µl injection volume). Retinas were harvested for analysis 120 and 240 days following transplantation. (**B, C**) The retinas isolated from post-transplantation were analyzed in flat-mounted tissue with confocal imaging. Transplanted human-induced pluripotent stem cell (hiPSC)-derived microglia were visualized through their expression of tdtomato (TdT) (**B**) or EGFP (**C**), while endogenous mouse microglia were visualized using immunostaining for mouse Tmem119 (mTmem119). Imaging analysis was performed in separate layers of the retina, including the ganglion cell layer (GL), inner plexiform layer (IPL), and outer plexiform layer (OPL). Scale bar = 100 µm. (**D**) The retinal section showed human iPSC-derived microglial cells integrated into whole retinal layers (top panel) and positively stained with human P2RY12 and TMEM119 microglia signature markers. Scale bar = 100 µm. The microglia cell number in GL, IPL, and OPL of host mouse retina were counted: mouse microglial cells (Iba1+, tdT−) and grafted human microglial cells (Iba1+, tdT+) were shown in (**E**), (**F**), and (**G**), respectively. ***p < 0.001, ****p < 0.0001, 3-6 biological replicates were performed. (**H**) and (**I**) showed tdT (**H**) or EGFP (**I**) labeled human iPSC-derived microglial cells in the IPL and OPL of the flat-mount retina with human CD11b staining. These results demonstrated that the infiltration of grafted hiPSC-derived microglial cells integrated into the mouse retina is general in nature and not cell line specific. Scale bar = 100 µm.

To assess the ability of hiPSC-derived microglia to serve as microglia donor cell sources for transplantation, we conducted xenotransplantation experiments using humanized immunodeficient Rag2^−/−^;IL2rg^−/−^;hCSF1^+/+^ transgenic mice as recipients as previously employed (Svoboda et al., 2019; Xu et al., 2020b; Chadarevian et al., 2023). Prior to xenotransplantation, recipient transgenic mice were pharmacologically depleted of endogenous retinal microglia by systemic administration of the CSF1R inhibitor PLX-5622 to create a depleted tissue niche for the integration of xenotransplanted microglia (Zhang et al., 2018). Two days following PLX-5622 treatment, adult transgenic mice were transplanted with 5000 hiPSC-derived microglia via injection into the subretinal space (Figure 5A). Tissue analysis at 4 and 8 months post-transplantation revealed that transplanted cells, which were marked by either tdTomato or EGFP expression, had migrated anteriorly from the subretinal space into the neural retina and were distributed across a wide retinal area within the inner and outer retinal layers including the ganglion cell layer (GCL), IPL, and OPL (Figure 5B–D) in retinal loci typically occupied by endogenous microglia. Transplanted cells were immunopositive for IBA1, and human CD11b (hCD11b) (Figure 5H, I), as well as for microglia signature markers hP2RY12 and hTMEM119 (Figure 5D). Interestingly, the transplanted cells within the retina showed a ramified morphology typical of homeostatic microglia and demonstrated a regularly tiled ‘mosaic’ distribution in their soma positions. These integrated cells were juxtaposed alongside residual endogenous murine microglia, which showed similar morphology and distribution as in endogenous conditions. This indicated that transplanted hiPSC-derived microglia responded to similar intraretinal cues and inter-microglia neighbor–neighbor interactions that guide the spatial and morphological organization of retinal microglia in vivo (Figure 5B–G). Similar observations were made for separate experiments involving the transplantation of tdtomato- and EGFP-expressing hiPSC-derived microglia (Figure 5B, C, H, I).

**Figure 5—figure supplement 1.**
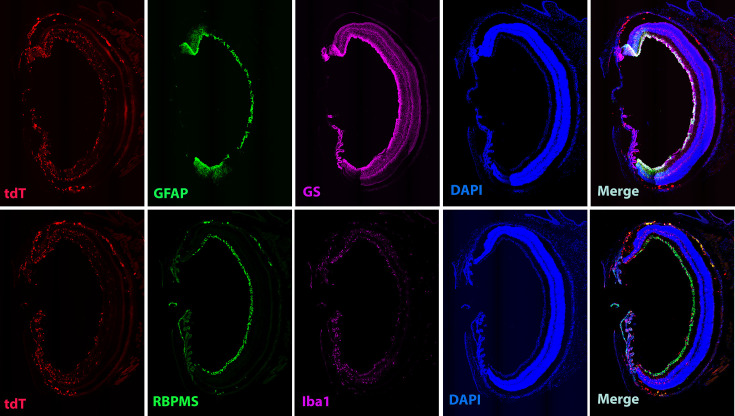
Homeostatic human-induced pluripotent stem cell (hiPSC)-derived microglial cells in the mouse retina do not affect local retinal cells. Entire section images showed GFAP, GS, RBPMS, and Iba1 staining for astrocytes, Müller cells, ganglion cells, and microglia cells in the retina after 4 months of xenotransplantation. Scale bar = 300 µm.

**Figure 5—figure supplement 2.**
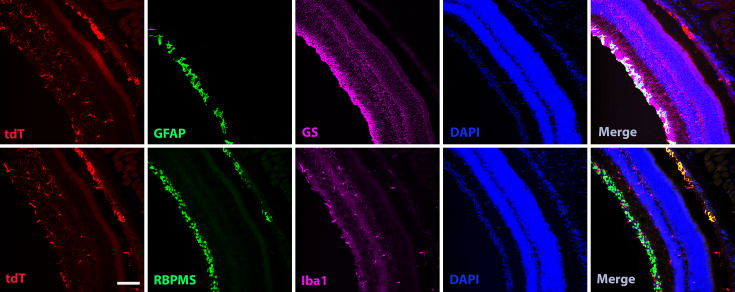
Homeostatic human-induced pluripotent stem cell (iPSC)-derived microglia cells in the mouse retina do not affect local retinal cells. The high-magnification images showed GFAP, GS, RBPMS, and Iba1 staining for astrocytes, Müller cells, ganglion cells, and microglia cells in the retina after 4 months of transplantation. Scale bar = 100 µm.

**Figure 5—figure supplement 3.**
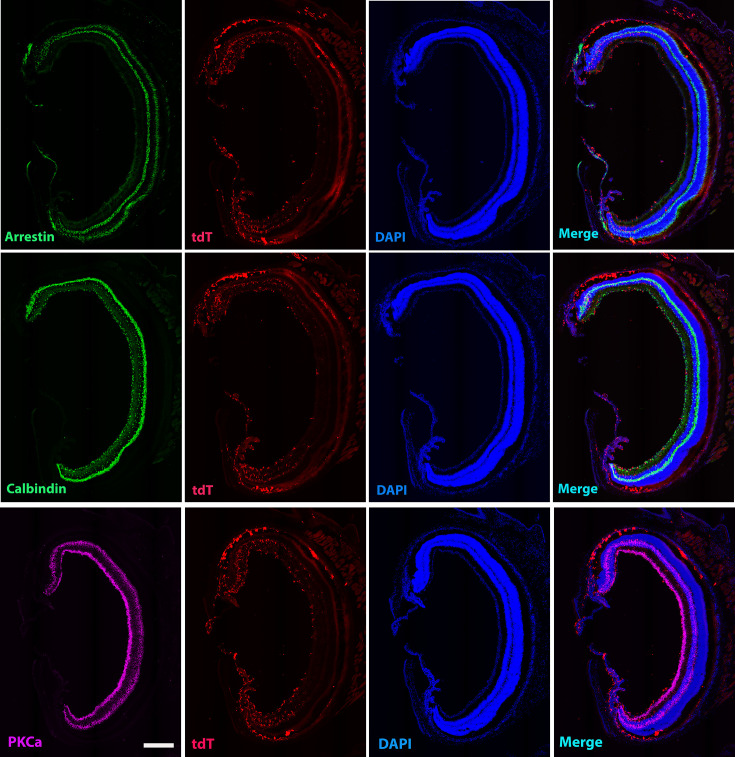
Homeostatic human-induced pluripotent stem cell (iPSC)-derived microglia cells in the mouse retina do not affect local retinal cells. Entire section images showed arrestin, calbindin, and PKCα staining for cone photoreceptors, horizontal and some amacrine cells, and bipolar cells in the retina after 4 months of xenotransplantation. Scale bar = 300 µm.

**Figure 5—figure supplement 4.**
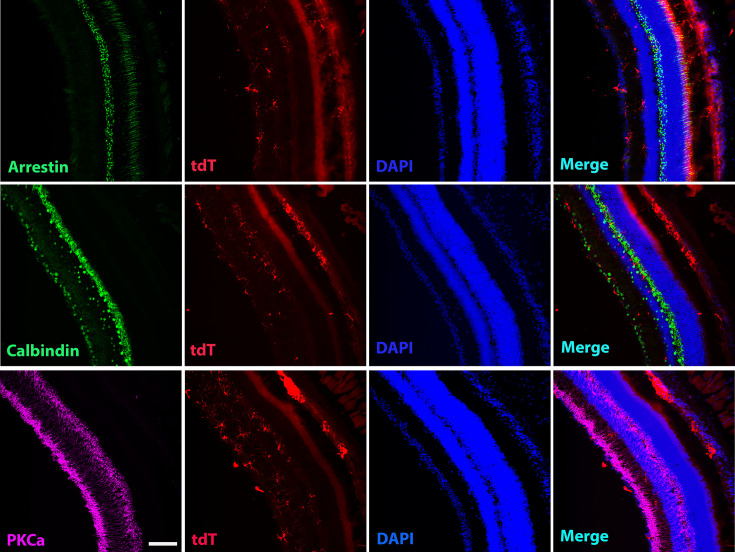
Homeostatic human-induced pluripotent stem cell (iPSC)-derived microglia cells in the mouse retina do not affect local retinal cells. The partial section of high-magnification images showed arrestin, calbindin, and PKCα staining for cone photoreceptors, horizontal and some amacrine cells, and bipolar cells in the retina after 4 months of xenotransplantation. Scale bar = 100 µm.

**Figure 5—figure supplement 5.**
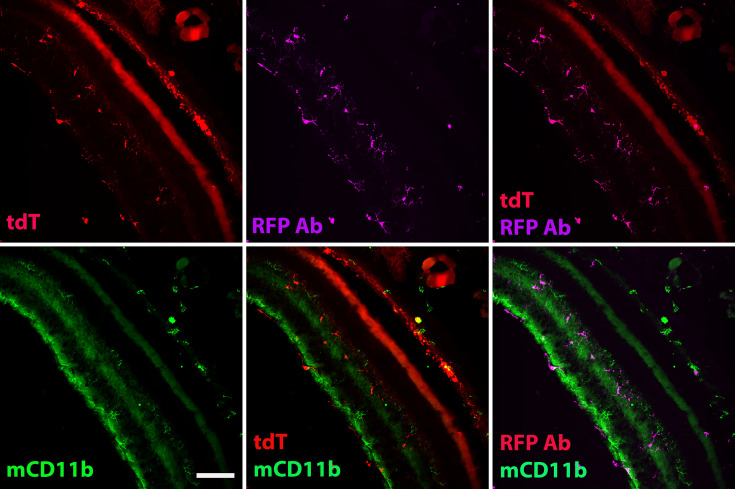
Homeostatic human-induced pluripotent stem cell (iPSC)-derived microglia cells in the mouse retina do not take over local retinal microglia cells. The section images showed Red Fluorescence Protein (RFP) and mouse CD11b staining to determine the tdT+ human microglia cells and local mouse microglia cells in the retina after 4 months of xenotransplantation. The results showed that the tdT+ human microglia cells colocalized with RFP staining (Far red) but not mouse CD11b (green) staining. Scale bar = 100 µm.

**Figure 5—figure supplement 6.**
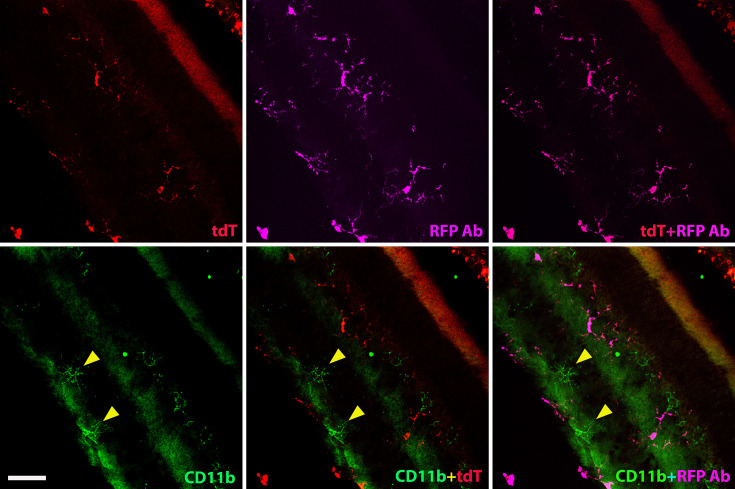
Homeostatic human-induced pluripotent stem cell (iPSC)-derived microglia cells in the mouse retina do not take over local retinal microglia cells. The hight magnification images showed RFP and mouse CD11b staining in the retina after 4 months of xenotransplantation. The results showed that the tdT+ human cells colocalized with RFP staining (Far red) but not mouse CD11b (green) staining. The triangle marker indicated that the local mouse microglia cells were only stained with mouse CD11b but not colocalized with tdT+ human microglia cells. Scale bar = 50 µm.

We further evaluated the impact of human iPSC-derived microglia xenotransplantation on the host mouse retinal cells (Figure 5—figure supplements 1–6). We examined the effect of microglia transplantation on endogenous Müller cell morphology and gliosis markers as retinal microglia have been described to interact with Müller glia to regulate overall retinal neuroinflammatory response (Wang et al., 2014). Immunostaining with glial fibrillary acidic protein (GFAP) and glutamine synthetase (GS) antibody (Figure 5—figure supplements 1 and 2) indicated that the transplanted human iPSC-derived microglial cells did not trigger any upregulation of Müller cell gliosis markers or induced morphological changes or reactive proliferation for up to 4 months post-transplantation. Additionally, the laminar organization of the inner and outer retinal layers remained normal and similar to those in control retinas not subjected to transplantation (Figure 5—figure supplements 1–4), indicating that microglia transplantation had no adverse impact on the structural integrity of the retina. Furthermore, immunostaining with the mouse CD11b antibody, which marked the residual population of endogenous mouse microglia showed the spatial juxtaposition of these mouse microglia with transplanted human iPSC-derived microglia in a common mosaic of tiled cells (Figure 5—figure supplements 5 and 6), indicating the ability of transplanted hiPSC-derived microglia to integrate with and exchange signals with the residual endogenous microglial population, but not replaced.

### Transplanted hiPSC-derived microglia respond to induction of RPE cell injury with migration and proliferation

To monitor the longer-term consequences of microglia xenotransplantation, we extended analysis and examined the location and morphology of transplanted hiPSC-microglia up to 8 months post-transplantation. Analysis of retinal flat mounts confirmed that the tdTomato+ cells remained appropriately located in the GCL, IPL, and OPL, forming a mosaic-like distribution typical of endogenous microglia (Figure 6—figure supplements 1 and 2). These cells maintained expression of hP2RY12 and hTMEM119, markers of homeostatic retinal microglia, indicating their long-term integration within the recipient mouse retina.

**Figure 6.**
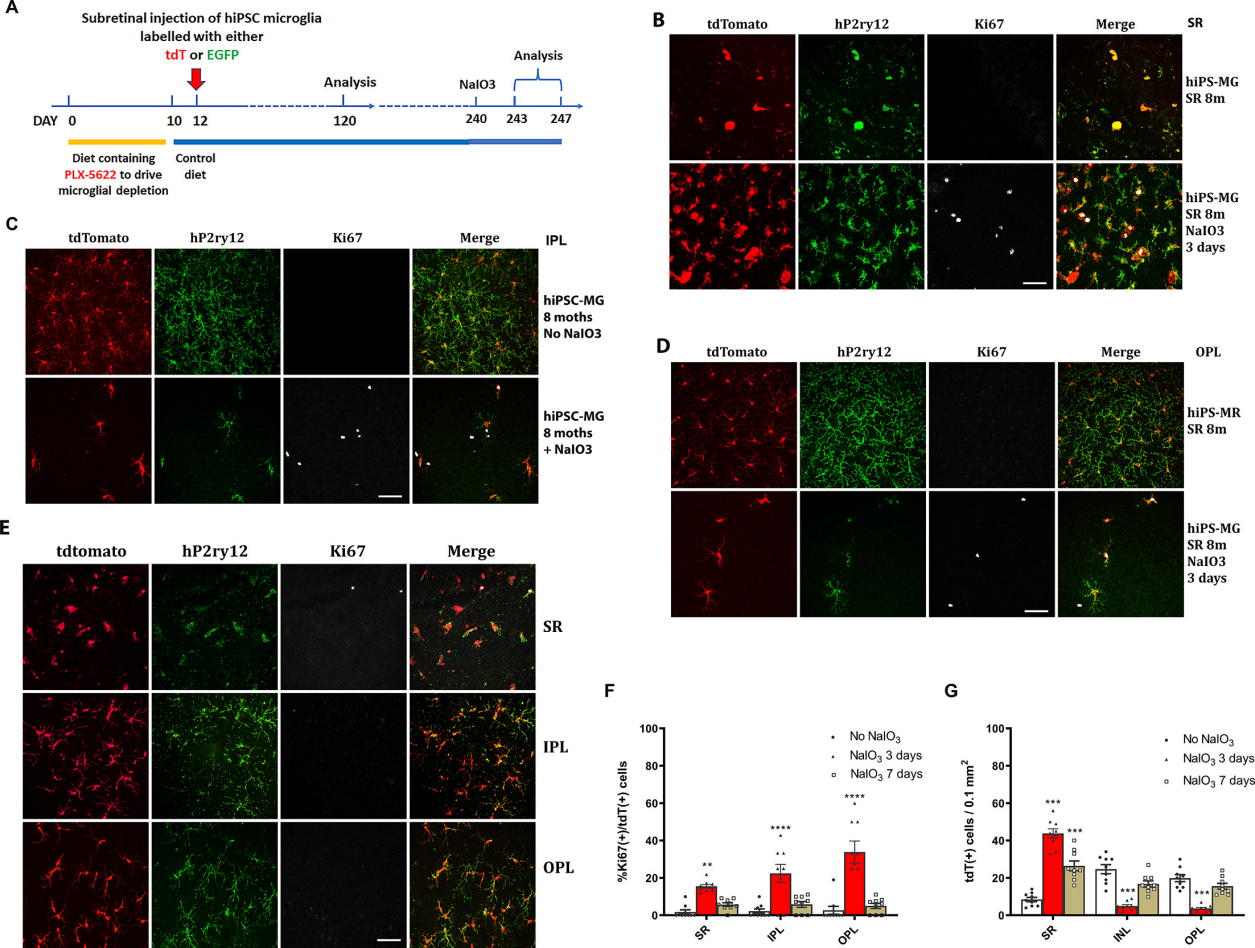
Migration and proliferation of human-induced pluripotent stem cell (hiPSC)-derived microglia in the mouse retina after sodium iodate (NaIO_3_)-induced retinal pigment epithelial (RPE) cell injury. (**A**) The schematic diagram shows the experiment’s procedure. After 8 months post-transplantation of hiPSC-derived microglia, recipient animals were administered NaIO_3_ (30 mg/kg body weight, intraperitoneal injection) to induce RPE injury. Retinas were harvested at 3 and 7 days after NaIO_3_ administration and microglia numbers in the retina and subretinal space will be monitored in retina and RPE-choroid flat mounts. (**B**) RPE-choroid flat mounts demonstrate an increase of hiPSC-derived microglia (tdTomato+ and P2RY12+) in the subretinal space in response to RPE injury. A subset of subretinal microglia labeled for Ki67 indicates active proliferation. Scale bar = 60 µm. (**C**) and (**D**) showed the number of P2RY12+ and tdtomato+ human microglial cells in inner plexiform layer (IPL) (**C**) and outer plexiform layer (OPL) (**D**) decreased; some of them showed Ki67+ staining, Scale bar = 60 µm. The cell count results showed in (**F**) and (**G**). (**E**) The retinal flat mount showed the number of P2RY12+ and tdtomato+ human microglial cells in IPL and OPL that were repopulated, and the cells stopped dividing with loss of the Ki67 staining at 7 days after NaIO_3_ injection. The cell numbers are shown in (**F**) and (**G**) (3 biological replicates). Scale bar = 60 µm, ** P<0.01, *** P<0.001, **** P,0.0001.

To evaluate the function of the transplanted hiPSC-derived microglia in the mouse retina and their ability to respond to injury in vivo, we subjected recipient mice 240 days post-transplantation to sodium iodate (NaIO_3_)-mediated RPE injury and analyzed retinal tissue 3 and 7 days post-injury (Figure 6A). In a previous study (Ma et al., 2017), we had characterized the responses of endogenous retinal microglia in this injury model; in the few days following injury, microglia within the neural retina migrated into the subretinal space, coming into close proximity to damaged RPE cells. This resulted in a transient decrease in microglia number in the IPL and OPL, which then recovered partially following the proliferation of the remaining microglia to replenish the depleted areas. We found that transplanted hiPSC-derived microglia demonstrated responses similar to endogenous microglia. Three days after NaIO_3_ injury, there was an increase in tdTomato+, hP2ry12+, hiPSC-derived microglia in the subretinal space, while their number decreased in the IPL and OPL (Figure 6B–D, G), indicating a migration of these cells from the neuroretina to the subretinal space. Some of the remaining tdTomato+ and P2ry12+ cells in the inner retina were positive for the cell-proliferation marker Ki67, indicating active cell division (Figure 6B–D, F). The numbers of Ki67+ tdTomato+ microglia peaked at 3 days post-injury and decreased thereafter (Figure 6B–F). Seven days post-injury, the numbers of tdTomato+ and P2ry12+ human iPSC-derived microglia increased in the IPL and OPL but decreased in the subretinal space (Figure 6E, G); Ki67+ tdTomato+ cell numbers also declined (Figure 6E, F). These findings suggest that once the hiPSC-derived microglia had replenished endogenous numbers in the inner retina, their division ceased. This response mirrors that of the endogenous mouse retinal microglia to NaIO_3_ injury (Ma et al., 2017).

**Figure 6—figure supplement 1.**
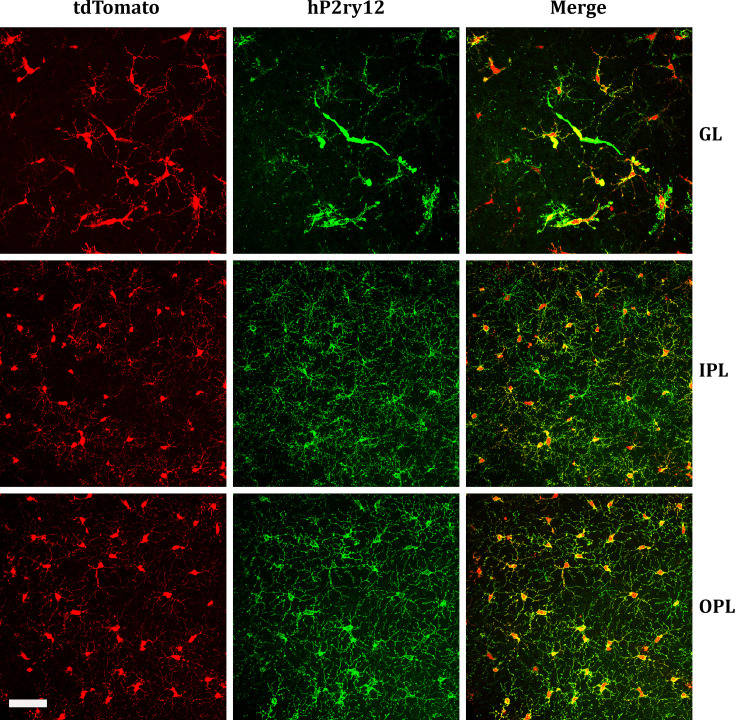
The images of hP2RY12 staining on the retina after 8 months of xenotransplantation. The images showed the tdtomato+ human microglia cells colocalized with hP2RY12 staining in GL, inner plexiform layer (IPL), and outer plexiform layer (OPL) in the mouse retina. Scale bar = 300 µm.

**Figure 6—figure supplement 2.**
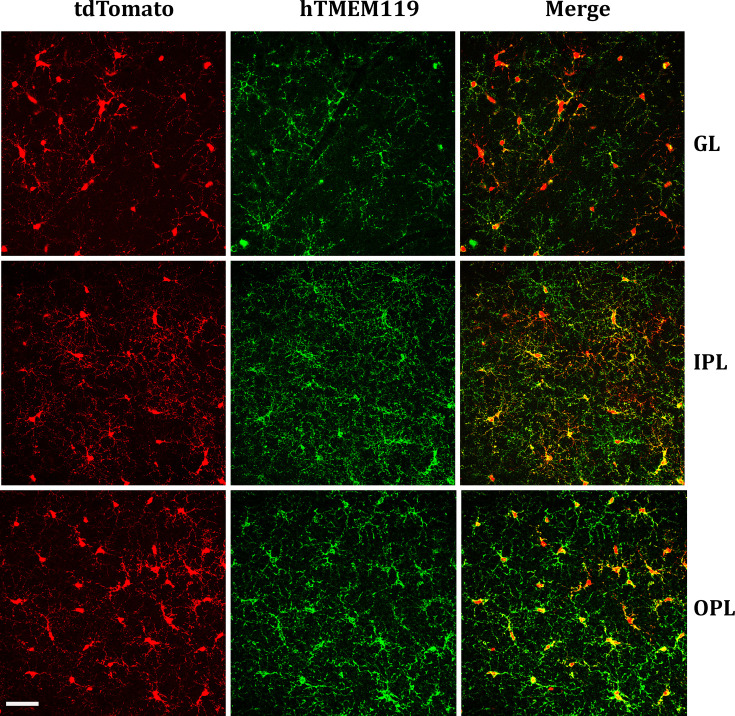
The images of hTMEM119 staining on the retina after 8 months of xenotransplantation. The images showed the tdtomato+ human microglia cells colocalized with hTMEM119 staining in GL, inner plexiform layer (IPL), and outer plexiform layer (OPL) in the mouse retina. Scale bar = 300 µm.

Overall, these results demonstrate that the transplanted human iPSC-derived microglial cells retained a capacity for migration and proliferative responses to injury in a manner observed for endogenous microglia.

### hiPSC-derived microglial cells phagocytize debris or dead photoreceptor cells after NaIO_3_-induced RPE cell injury

**Figure 7.**
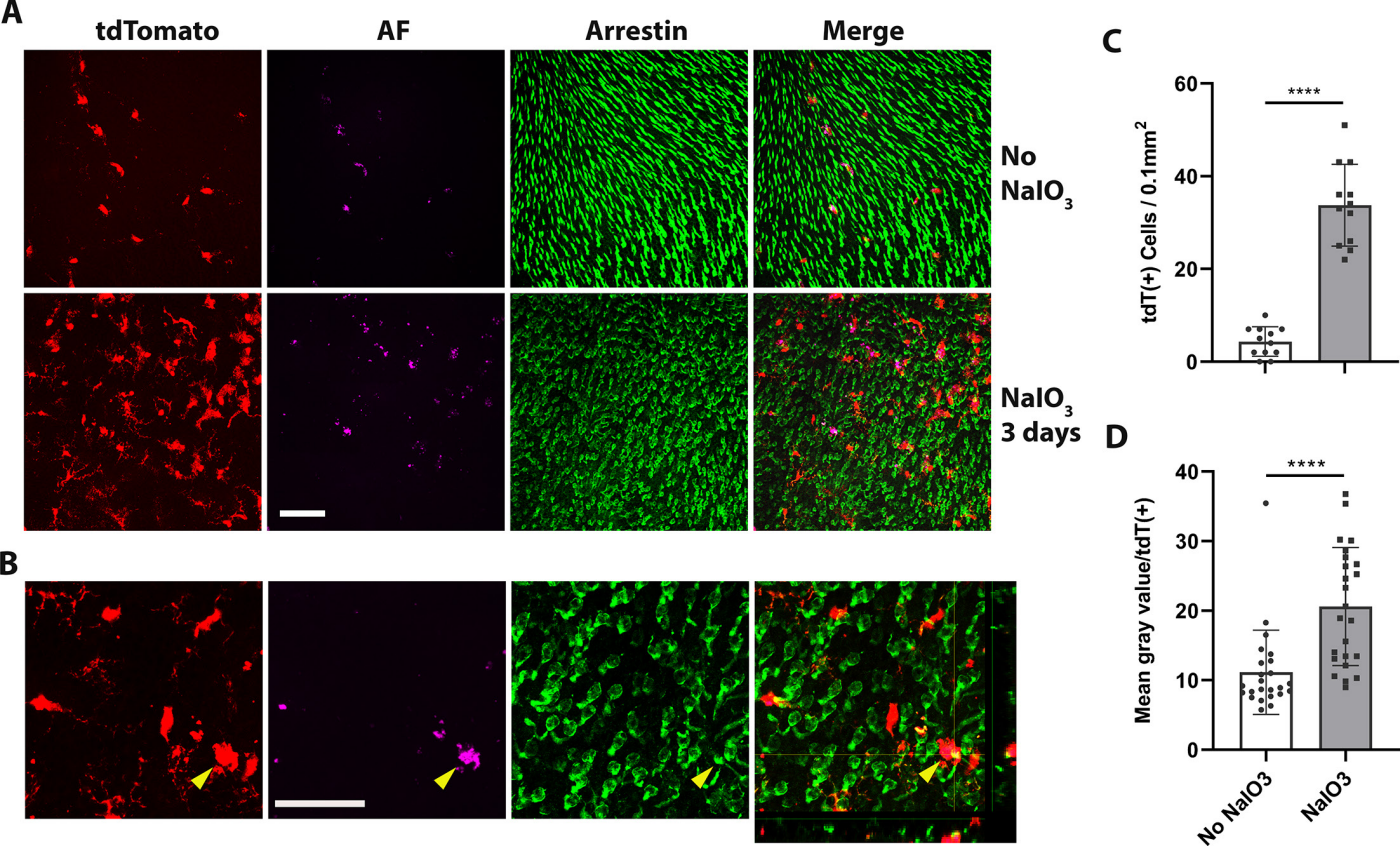
Dyshomeostatic human-induced pluripotent stem cell (iPSC)-derived microglial cells in the mouse retina phagocytose dead photoreceptor cells/debris after retinal pigment epithelial (RPE) cell injury. (**A**) Dyshomeostatic human microglial cells (tdtomato+) accumulated in the photoreceptor cell layer after 3 days of sodium iodate (NaIO_3_)-induced RPE cell injury compared with no NaIO_3_ administration. The photoreceptor cells stained with cone arrestin (green) and autofluorescence showed in magenta. Scale bar = 60 µm. (**B**) High-magnificent images and the side view showed human microglial cells (red) co-labeled with photoreceptor cells arrestin staining (green) after 3 days of NaIO_3_ injury. The yellow arrowhead showed the colocalized tdT+ human microglia cell and arrestin+ cone photoreceptor cell. Scale bar = 40 µm. (**C**) The number of tdtomato+ human microglial cells in the photoreceptor layer. (**D**) The mean gray autofluorescence value in each human microglia cell. ****p < 0.0001.

Phagocytosis, a critical function of microglia, is essential both during development and in the resolution of pathological processes. Retinal microglia adaptively phagocytose and clear apoptotic photoreceptors in the rd10 mouse model of photoreceptor degeneration (Silverman et al., 2019). We found here that 3 days following NaIO_3_-induced RPE cell injury, tdTomato+ transplanted hiPSC-derived microglia migrated not only to the subretinal space but also into the photoreceptor layer (Figure 7A–C), coincident with the time of photoreceptor degeneration, when photoreceptor morphology becomes disrupted and photoreceptor density decreases (Figure 7A). Within the photoreceptor layer, hiPSC-derived microglia were observed to phagocytose photoreceptors as evidenced by the accumulation of intracellular autofluorescent material in their soma (Figure 7A, B, D), and their transformation into larger amoeboid cells (Figure 7B, yellow arrowhead) containing arrestin-immunopositive photoreceptor-derived debris. mRNA analysis for human-specific transcripts also indicated that transplanted hiPSC-derived microglia upregulated inflammatory cytokine expression, increased phagocytosis, and decreased expression of microglia homeostatic genes and neurotrophic factors (Figure 7—figure supplement 1; Supplementary file 5). Taken together, these findings further demonstrate that the xenografted human iPSC-derived microglial cells generate functional responses to in vivo injury that closely resemble those of endogenous homeostatic retinal microglia.

**Figure 7—figure supplement 1.**
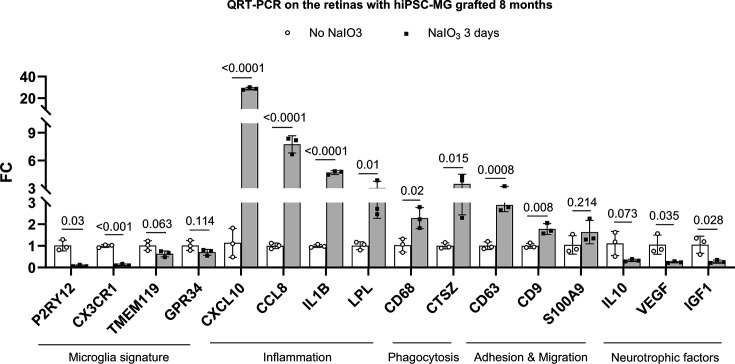
The inflammation, phagocytosis, adhesion and migration, neurotrophic factors, and microglia signature gene expression in human-induced pluripotent stem cell (hiPSC)-derived microglia cells of grafted retinas. The gene coding sequences were compared between humans and mice, a human-specific sequence was chose to make the oligos (Supplementary file 5), and ran a quantitative reverse transcription-PCR (qRT-PCR) on 8-month hiPSC-derived microglia cells grafted retinas with/without sodium iodate (NaIO_3_)-treated retinas. The results revealed that hiPSC-derived microglia cells expressed more inflammatory factors and phagocytosis genes and promoted cell migration but decreased microglia cell signature genes and neurotrophic factors in NaIO_3_-treated retina (3 biological replicates in each group).

## Discussion

**Figure 8.**
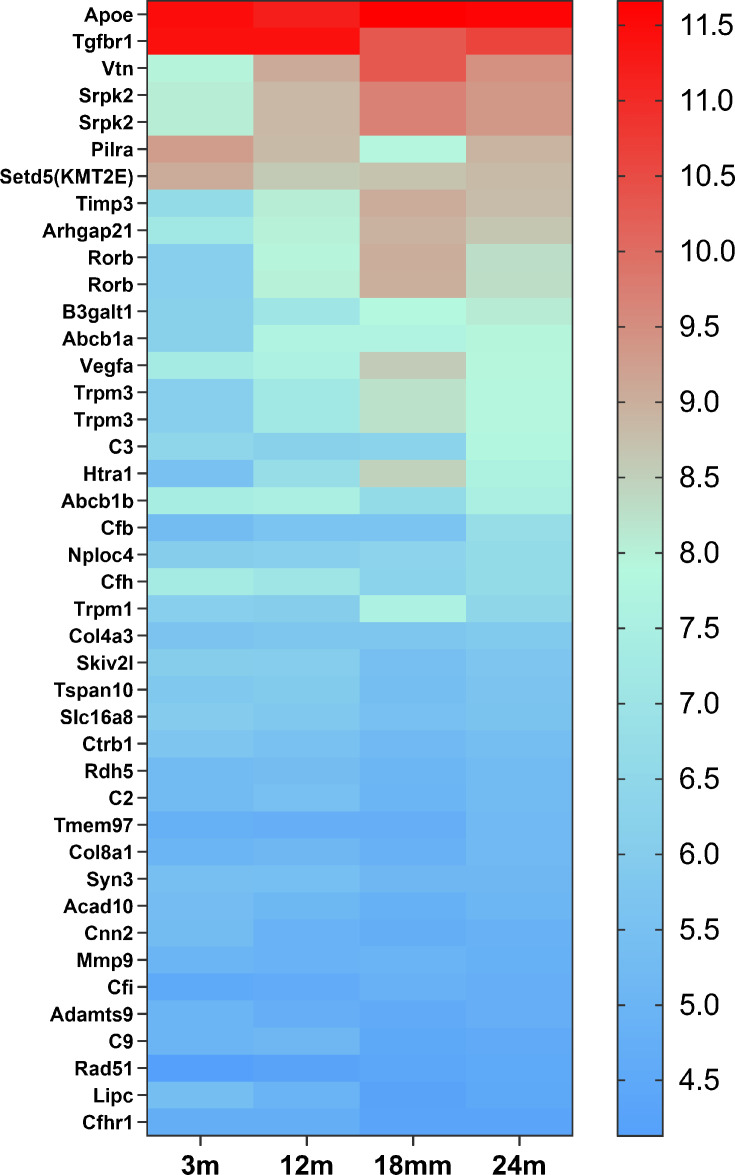
The heat map of 42 candidate genes from 34 loci associated with age-related macular degeneration (AMD) expressed in retinal microglia cells. The microglia gene expression data are from microarray data previously published (Ma et al., 2013). The candidate genes came from the published paper (den Hollander et al., 2022). The gene list is in Supplementary file 6.

**Figure 9.**
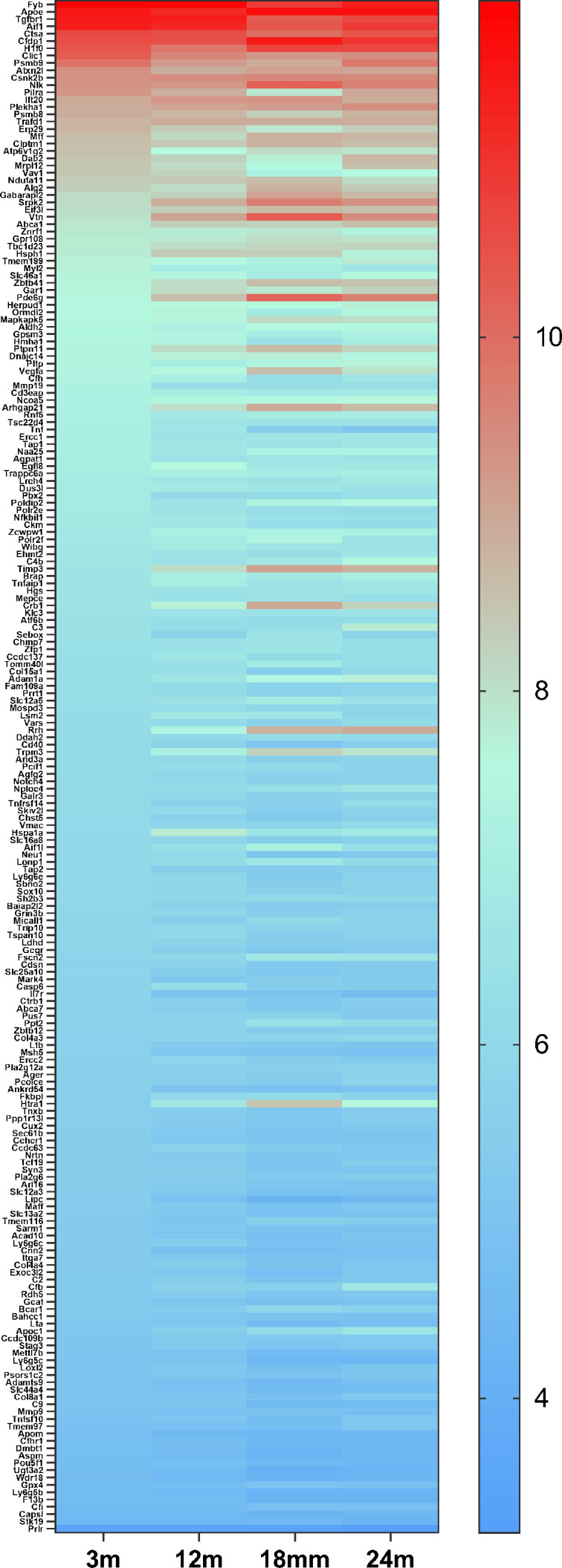
The heat map of 209 genes associated with age-related macular degeneration (AMD) (Fritsche et al., 2016) expressed in retinal microglia cells. The microglia gene expression data are from microarray data previously published (Ma et al., 2013). The gene list is in Supplementary file 7.

Microglial cells are instrumental in the development and progression of numerous CNS diseases. For instance, in AD, microglia are enriched in over 50% of associated gene loci implicated in AD risk (Hasselmann and Blurton-Jones, 2020). Similarly, in AMD, 57% of 368 genes, located close to 52 AMD gene loci, are expressed in retinal microglial cells (Figure 9), and 52% of them are highly expressed in microglial cells (Figures 8 and 9; Fritsche et al., 2016; Ma et al., 2013; den Hollander et al., 2022). Understanding microglia cell functions is essential for investigating disease mechanisms and identifying accurate therapeutic targets. Most of our current knowledge about microglial cells comes from rodent studies. However, genetic and functional differences exist between murine and human microglia (Galatro et al., 2017; Gosselin et al., 2017; Smith and Dragunow, 2014). Hence, more in-depth knowledge of human microglial cells in vitro and in vivo is required.

Human iPSCs offer promising prospects for many retinal research fields (Zhong et al., 2014; Leach et al., 2016; Tanaka et al., 2016). For over a decade, macrophage/microglial cells have been differentiated using human iPSC (Karlsson et al., 2008; Pocock and Piers, 2018). An abundance of hiPSC-derived microglial cells, with defined genomic background and easy manipulation of hiPSC, offer substantial benefits in various research areas.

Under in vivo physiological conditions, microglial cells exhibit a tile-like arrangement, without overlap. They try to maintain this property in culture, allowing overgrown cells to float out to the medium. Based on this phenomenon and a variety of established microglia cell differentiation protocols (Muffat et al., 2016; Pandya et al., 2017; Abud et al., 2017; Douvaras et al., 2017; Haenseler et al., 2017; Takata et al., 2017), We chose to employ the myeloid progenitor/microglia cell floating culture method (described in van Wilgenburg et al., 2013; Haenseler et al., 2017) due to its simplicity, efficiency, and consistency. This approach facilitates the generation of a large and uniform population of myeloid progenitor cells, with over 98.6% expressing the *CD34* marker, sustained over a 3-month period. These progenitor cells differentiate into pure microglial cells (>98.5% *P2RY12+*), bearing a profile rich in microglia genes, and demonstrate characteristics similar to native microglia in physiological CNS tissue. A comparison of the signaling pathway to myeloid cells reveals the central hubs of signaling as IL6, IL1b, and the stat3 pathway (Figure 2D), which are key to microglia functioning during inflammation. The differentiation protocol employing floating myeloid progenitor cells produces a significant number of CNS resident-like microglial cells. These hiPSC-derived microglial cells respond robustly to LPS stimulation and demonstrate phagocytic activity, mimicking primary cultured retinal microglial cells. Gene expression profile comparison between hiPSC-microglial cells and fetal/adult brain microglia revealed that hiPSC-derived microglial cells are comparable to fetal and adult microglial cells but are far away from monocytes and inflammatory monocytes (Figure 2—figure supplements 2–4; Supplementary files 2 and 3). Therefore, they effectively replicate resident microglia characteristics (Ulland and Colonna, 2018; Wang et al., 2020; Shi et al., 2022; Guo et al., 2022).

CNS disease treatment strategies include gene regulation, gene delivery (Neumann, 2006; Beutner et al., 2013), rejuvenation (Karlstetter et al., 2015; Elmore et al., 2018), or replacement of dysfunctional microglial cells (Willis et al., 2020; Xu et al., 2020a; Han et al., 2020; Shibuya et al., 2022). hiPSC-derived microglial cells offer a potentially unlimited source for cell replacement therapy. The in vivo functionality of these cells was verified through xenografting into adult Rag2−/−; IL2rg−/−;hCSF1+/+ mice. The grafted microglial cells integrated into the right location of the retina, expressing microglia signature genes and formed new homeostasis with resident microglial cells for 8 months. Homeostatic hiPSC-derived microglia responded to RPE cell injury like host retinal microglial cells, marking the success of the xenotransplantation model (Guilliams and Scott, 2017) and highlighting the potential for hiPSC-derived microglial cells in retinal microglia cell replacement therapy, such as in AMD.

Several exogenous microglia cell replacement techniques have been investigated, with these methods varying based on the type of donor cells used and the recipient’s age. Initial microglia cell replacement studies started from the transplantation of hematopoietic stem cells (HPSCs) (Larochelle et al., 2016; Xu et al., 2020b; Hohsfield et al., 2020). However, even after differentiation in local tissue, HPSC-derived microglial cells maintained some gene expressions distinct from the original resident microglial cells (Lund et al., 2018), warranting further exploration of these HPSC-derived microglial cells’ unique characteristics. Another method involves using newborn mice as recipients, with iPSC or stem cell-derived microglial cells as the donors (Mancuso et al., 2019; Xu et al., 2020a). This approach does not require the depletion of resident microglial cells, and the grafted cells can infiltrate the brain tissue, distributing similarly to the original resident microglial cells. While suitable for examining microglia cell function in various backgrounds, the clinical applicability of this method remains limited.

A third transplantation approach involves adult recipients receiving iPSC or stem cell-derived microglial cells after resident microglia cell depletion (Chadarevian et al., 2023). Since this technique requires a microglial-empty niche, resident microglia must be depleted or relocated from the retina, typically done by using CSF1R inhibitors. To prevent these inhibitors from affecting the grafted cells, investigators have tried using modified CSF1R microglial cells as donor cells (Chadarevian et al., 2023). In our research, we found a 2-day recovery period with normal food intake sufficient to clear the CSF1R inhibitor, allowing grafted cells to integrate into microglia empty niche. In this study, we used the subretinal xenotransplantation method, though alternative transplantation routes such as intravenous injection, vitreous, and suborbital space delivery warrant further exploration.

In conclusion, hiPSCs can be differentiated into microglial cells through a simplified common pathway and factors, although the more precise differentiation factors still need further investigation under in vitro conditions. For instance, the microglia signature gene, TMEM119, shows low expression in hiPSC-derived microglia single-type cell culture conditions. Interestingly, we discovered that a medium composed of a mix of cultured EBs and microglia progenitor cells fosters further differentiation of microglial cells. The humanized mouse model established through xenotransplantation serves as a reliable tool for studying the functions of various hiPSC-derived microglial cells. This model offers a valuable platform for investigating disease mechanisms and evaluating the therapeutic effects of xenografted hiPSC-derived microglial cells from variety of backgrounds.

## Materials and methods

**Key resources table:** 

Reagent type (species) or resource	Designation	Source or reference	Identifiers	Additional information
Genetic reagent (*M. musculus*)	*C;129S4-Rag2tm1.1Flv Csf1tm1(CSF1)Flv Il2rgtm1.1Flv/J*	PubMed:21791433	MGI:J:177073	RRID:IMSR_JAX:017708
Genetic reagent (*Homo sapiens*)	*KYOUDXR0109B*	ATCC	ACS-1023	Human-induced pluripotent stem cells (iPSCs)
Genetic reagent (*Homo sapiens*)	*NCRM6*	NHLBI	NCRM6 (female) iPSC line	From CD34^+^ cells, Episomal vectors
Genetic reagent (*Homo sapiens*)	*MS19-ES-H*	NHLBI	MS19-ES-H (female) iPSC line	From PBMS cells, Cytotune Sendai Virus kit
Genetic reagent (*Homo sapiens*)	*NCRM5-AAVS1-CAG-EGFP*	NHLBI	NCRM5-AAVS1-CAG-EGFP (clone 5)	From CD34^+^ cells, NCRM5 (male) reporter iPSC line with CAG-EGFP targeted mono-allelically at AAVS1 safe harbor
Genetic reagent (*Homo sapiens*)	*ND2-AAVS1-iCAG-tdTomato*	NHLBI	ND2-AAVS1-iCAG-tdTomato (clone 1)	From fibroblast cells, ND2 (male) reporter iPSC line with insulated CAG-tdTomato targeted mono-allelically at AAVS1 safe harbor
Antibody	anti-Iba1 (rabbit polyclonal)	Wako	Cat. #: 019-19741, RRID:AB_839504	IHC (1:500)
Antibody	anti-human TMEM119 (rabbit polyclonal)	Sigma-Aldrich	Cat. #: HPA051870, RRID:AB_2681645	IHC (1:100)
Antibody	anti-human CD68 (mouse monoclonal)	R&D	Cat. #: MAB20401	IHC (1:100)
Antibody	anti-human CD45 (mouse monoclonal)	R&D	Cat. #: FAB1430R	IHC (1:100)
Antibody	anti-human CD11b (mouse monoclonal)	R&D	Cat. #: FAB1699R	IHC (1:100)
Antibody	anti-human CX3CR1 (rat monoclonal)	Invitrogen	Cat. #: 61-6099-42	IHC (1:100)
Antibody	anti-human HLA (mouse monoclonal)	Invitrogen	Cat. #: 11-9983-42	IHC (1:100)
Antibody	anti-mouse CD11b (rat monoclonal)	Bio-Rad	Cat. #: MCA711G	IHC (1:100)
Antibody	anti-GFAP (rat monoclonal)	Invitrogen	Cat. #: 13-0300, RRID:AB_2532994	IHC (1:200)
Antibody	anti-mouse TMEM119 (guinea pig polyclonal)	Synaptic systems	Cat. #: 400 004, RRID:AB_2832239	IHC (1:500)
Antibody	anti-CD68 (rat monoclonal)	Bio-Rad	Cat. #: MCA1957, RRID:AB_322219	IHC (1:200)
Antibody	anti-CD34 (rat monoclonal)	eBioscience	Cat. #: 14-0341	IHC (1:30)
Antibody	anti-PU.1 (rabbit monoclonal)	Thermo Fisher Scientific	Cat. #: MA5-15064	IHC (1:200)
Antibody	anti-Trem2 (rabbit monoclonal)	Thermo Fisher Scientific	Cat. #: 702886	IHC (1:100)
Antibody	anti-CD45 (rat monoclonal)	Bio-Rad	Cat. #: MCA1388, RRID:AB_321729	IHC (1:100)
Antibody	anti glutamine synthetase (mouse monoclonal)	Millipore	Cat. #: MAB302, RRID:AB_2110656	IHC (1:200)
Antibody	anti-RBPMS (guinea Pig polyclonal Ab)	Phosphosolutions	Cat. #: 1832-RBPMS	IHC (1:100)
Antibody	anti-cone arrestin (rabbit polyclonal)	Millipore	Cat. #: AB15282, RRID:AB_1163387	IHC (1:200)
Antibody	anti-calbindin (rabbit polyclonal)	Swant	Cat. #: CB-38a	IHC (1:5000)
Antibody	anti-PKCa (rabbit polyclonal)	Sigma-Aldrich	Cat. #: P4334, RRID:AB_477345	IHC (1:200)
Antibody	anti-RFP (rabbit polyclonal)	RockLand	Cat. #: 600-401-379-RTU	IHC (1:100)
Antibody	anti-Ki67-660 (rat monoclonal)	eBioscience	Cat. #: 50-5698-82, RRID:AB_2574235	IHC (1:50)
Antibody	anti-P2RY12 (rabbit polyclonal)	Thermo Fisher Scientific	Cat. #: PA5-77671, RRID:AB_2736305	IHC (1:100)
Antibody	anti-P2RY12 (rabbit polyclonal)	Sigma-Aldrich	Cat. #: HPA014518, RRID:AB_2669027	IHC (1:100)
Antibody	Goat anti-Rabbit IgG Alexa Fluor 488	Invitrogen	Cat. #: A27034, RRID:AB_2536097	IHC (1:200)
Antibody	Goat anti-Rabbit IgG Alexa Fluor 568	Invitrogen	Cat. #: A11011, RRID:AB_143157	IHC (1:200)
Antibody	Goat anti-Rabbit IgG Alexa Fluor 647	Invitrogen	Cat. #: A32733, RRID:AB_2633282	IHC (1:200)
Antibody	Goat anti-mouse IgG Alexa Fluor 488	Invitrogen	Cat. #: A28175, RRID:AB_2536161	IHC (1:200)
Antibody	Goat anti-mouse IgG Alexa Fluor 568	Invitrogen	Cat. #: A-11004, RRID:AB_141371	IHC (1:200)
Antibody	Goat anti-mouse IgG Alexa Fluor 647	Invitrogen	Cat. #: A-21235, RRID:AB_141693	IHC (1:200)
Antibody	Donkey anti-Rat IgG Alexa Fluor 488	Invitrogen	Cat. #: A-21208, RRID:AB_141709	IHC (1:200)
Antibody	Donkey anti-Rat IgG Alexa Fluor 594	Invitrogen	Cat. #: A-21209, RRID:AB_2535795	IHC (1:200)
Antibody	Donkey anti-Rat IgG Alexa Fluor 650	Invitrogen	Cat. #: SA5-10029, RRID:AB_2556609	IHC (1:200)
Antibody	Rat monoclonal anti CD11b, Alexa Fluor 488	eBioscience	Cat. #: 53-0112-82, RRID:AB_469901	IHC (1:50)
Peptide, recombinant protein	human M-CSF	Invitrogen	Cat. #: PHC9501	
Peptide, recombinant protein	Human IL3	R&D	Cat. #: 203-IL-100	
Peptide, recombinant protein	human IL-34	Peprotech	Cat. #: 200–34	
Peptide, recombinant protein	human CX3CL1	Peprotech	Cat. #: 300–31	
Peptide, recombinant protein	human TGFb1	R&D	Cat. #: 7666-MB-005	
Peptide, recombinant protein	human TGFb2	R&D	Cat. #: 7346-B2-005	
Peptide, recombinant protein	human BMP-4	GIBCO	Cat. #: PHC9534	
Peptide, recombinant protein	human SCF	Miltenyi Biotec	Cat. #: 130096692	
Peptide, recombinant protein	human VEGF	GIBCO	Cat. #: PHC9394	
Chemical compound	PLX5622	Plexxikon	PLX5622 was provided by Plexxikon Inc and formulated in AIN-76A standard chow by Research Diets Inc	1200 mg/kg in chow
Chemical compound	NaIO_3_	Sigma-Aldrich	Cat. #: S4007	
Chemical compound	BSA	Sigma-Aldrich	Cat. #: A2153	
Chemical compound	FBS	Thermo Fisher Scientific	Cat. #: A3160702	
Chemical compound	Ketamine	Anased	Cat. #: NDC13985-584-10	
Chemical compound	Xylazine	Anased	Cat. #: NDC59399-110-20	
Chemical compound	Topical tropicamide	Alcon	Cat. # 215340	
Chemical compound	Phenylephrine	Alcon	Cat. l# 215664	
Chemical compound	0.5% Proparacaine HCL	Sandoz	Cat. #: 101571	
Chemical compound	Surcrose	Sigma-Aldrich	Cat. #: S7903-5KG	
Chemical compound	OCT	Thermo Fisher Scientific	Cat. #: 23-730-571	
Chemical compound	Fluorescein AK-FLUOR	Akorn	Cat. #: 17478-253-10	
Chemical compound	Tamoxifen	Sigma-Aldrich	Cat. #: T5648-5G	
Chemical compound	HBSS	Sigma-Aldrich	Cat. #: H8264-1L	
Chemical compound	L-(+)-Cysteine hydrochloride monohydrate	Fisher	Cat. #: C562-25	
Chemical compound	Papain, lyophilized	Worthington Biochemical	Cat. #: LS003119	
Chemical compound	DNAse I	Worthington Biochemical	Cat. #: LS006333	
Chemical compound	Superoxide dismutase	Worthington Biochemical	Cat. #: LS003540	
Chemical compound	Catalase	Sigma-Aldrich	Cat. #: C1345-1G	
Chemical compound	(+)-α-Tocopherol acetate	Sigma-Aldrich	Cat. #: T-1157-1G	
Chemical compound	Gentamicin solution	Sigma-Aldrich	Cat. #: G1397-10ml	
Chemical compound	D-(+)-Glucose	Sigma-Aldrich	Cat. #: G7021-100g	
Chemical compound	Antipain dihydrochloride	Roche	Cat. #: 11004646001	
Chemical compound	HEPES	Invitrogen	Cat. #: 15630080	
Chemical compound	EDTA	KD medical	Cat. #: RGC-3130	
Chemical compound	RNAlater solution	Ambion	Cat. #: AM7021	
Chemical compound	Triton X-100	Sigma-Aldrich	Cat. #: X100100ml	
Chemical compound	Tween 20	Sigma-Aldrich	Cat. #: P1379-100ml	
Chemical compound	Paraformadehyde	Fisher Scientific	Cat. #: 50-259-97	
Chemical compound	Donkey serum	Sigma-Aldrich	Cat. #: D9663-10ml	
Chemical compound	Goat serum	Sigma-Aldrich	Cat. #: G9023-10ml	
Commercial assay or kit	Bloking Reagent	Sigma-Aldrich	Cat. #: 11096176001	
Commercial assay or kit	In Situ Cell Death Detection Kit, TMR red	Sigma-Aldrich	Cat. #: 12156792910	
Commercial assay or kit	Ib4 Alexa Fluor 568	Invitrogen	Cat. #: I21412	IHC (1:200)
Commercial assay or kit	Ib4 Alexa Fluor 647	Invitrogen	Cat. #: I32450	IHC (1:200)
Commercial assay or kit	DAPI	Sigma-Aldrich	Cat. #: D9542	IHC (1:200)
Commercial assay or kit	Mounting medium without DAPI	Vector	Cat. #: H-1000	
Commercial assay or kit	Mounting medium with DAPI	Vector	Cat. #: H-1200	
Commercial assay or kit	RNeasy Mini Kit	QIAGEN	Cat. #: 74104	
Commercial assay or kit	Rnase free Dnase set	QIAGEN	Cat. #: 79254	
Commercial assay or kit	First strand cDNA synthesis	Takara	Cat. #: 6110A	
Commercial assay or kit	MessageBooster cDNA synthesis kit	Epicentre	Cat. #: MB060110	
Commercial assay or kit	Fast SYBR Green Master Mix	Thermo Fisher Scientific	Cat. #: 4385617	
Commercial assay or kit	LiDirect Lightening genotyping kit	LifeSci	Cat. #: M0015	
Commercial assay or kit	eBioscience Flow Cytometry Staining Buffer	Thermo Fisher Scientific	Cat. #: 00-4222-57	
Commercial assay or kit	X-VIVO-15 medium	Lonza	Cat. #: BEBP02-061Q	
Commercial assay or kit	DMEM:F12 medium	Thermo Fisher Scientific	Cat. #: 11330057	
Commercial assay or kit	mTeSR1	Stemcell technologies	Cat. #: 85850	
Commercial assay or kit	N2 supplement	Thermo Fisher Scientific	Cat. #: 17502048	
Commercial assay or kit	Non-essential Amino Acids (NEAA)	Thermo Fisher Scientific	Cat. #: 11140050	
Commercial assay or kit	GlutaMax Supplement	Thermo Fisher Scientific	Cat. #: 35050061	
Commercial assay or kit	Geltrex	Thermo Fisher Scientific	Cat. #: A1413301	
Commercial assay or kit	TrypLE Express	Thermo Fisher Scientific	Cat. #: 12605010	
Commercial assay or kit	Rho-kinase inhibitor Y-27632	abcam	Cat. #: ab143784	
Commercial assay or kit	mFreSR	Stemcell technologies	Cat. # 05854	
Commercial assay or kit	Stem Cell Dissociation Reagent	ATCC	Cat, #: ACS-3010	
Commercial assay or kit	Stem Cell Freezing Media	ATCC	Cat. #: ACS-3020	
Commercial assay or kit	Penicillin-Streptomycin	Thermo Fisher Scientific	Cat. #: 15140122	
Commercial assay or kit	pHrodo Red *E. coli* BioParticles	Thermo Fisher Scientific	Cat. #: P35361	
Commercial assay or kit	pHrodo Red Zymosan Bioparticles	Thermo Fisher Scientific	Cat. #: P35364	
Commercial assay or kit	Bovine rod outer segment	Invision Bioresources	Cat. #: 98740	
Commercial assay or kit	Vybrant DiI Cell-Labeling Solution	Thermo Fisher Scientific	Cat. #: V22885	
Commercial assay or kit	Lipopolysaccharides (LPS)	Sigma-Aldrich	Cat. #: L2630	
Commercial assay or kit	RIPA lysis buffer	Sigma-Aldrich	Cat. #: R0278	
Commercial assay or kit	proteinase inhibitor mixture	Calbiochem	Cat. #: 539132	
Commercial assay or kit	Pierce BCA Protein Assay Kit	Thermo Fisher Scientific	Cat. #: 23227	
Commercial assay or kit	Milliplex bead assay kit	Millipore	Cat. #: MCYTOMAG-70K	
Commercial assay or kit	AggreWellsTM800	Stemcell technologies	Cat. #: 34825	
Software	ImageJ	ImageJ (http://imagej.nih.gov/ij/)	RRID:SCR_003070	
Software	GraphPad Prism7	GraphPad Prism (https://graphpad.com)	RRID:SCR_015807	Version 7
Software	IPA	QIAGEN	RRID:SCR_008653	
Software	JMP	JMP	RRID:SCR_014242	Version 12

### Experimental animals and PLX-5622 treatment

In vivo experiments were conducted according to protocols (NEI-698) approved by the Institutional Animal Care and Use Committee (National Eye Institute Animal Care and Use Committee) and adhered to the Association for Research in Vision and Ophthalmology (ARVO) statement on animal use in ophthalmic and vision research. Rag2^−/−^;IL2rg^−/−^;hCSF1^+/+^ transgenic mice were obtained from Jackson Laboratories (Stock #17708). Animals were housed in a National Institutes of Health (NIH) animal facility under a 12-hr light/dark cycle and fed standard chow. Genotype analysis by sequencing revealed that the rd8 mutation was absent in the *Crb1* gene. To deplete retinal microglia, 2-month-old experimental animals were administered a diet containing PLX-5622 (Plexxikon, at 1200 parts per million), a potent and selective inhibitor of the CSF1R previously demonstrated to deplete the majority of microglia in the mouse brain (Dagher et al., 2015) and retina (Zhang et al., 2018). Animals were maintained continuously on the PLX-5622 diet for 10 days and then switched back to standard chow.

### Human iPSC culture

Five human iPSC lines were used in this study. The KYOUDXR0109B hiPSC line was generated in Yamanaka Lab from fibroblasts isolated from a healthy female donor and reprogrammed by the expression of OCT4, SOX2, KLF4, and MYC using retroviral transduction. Cells are tested for postfreeze viability and growth, sterility (including mycoplasma), identity by short-tandem repeat (STR) analysis and karyotype by G-banding. Each lot is tested for pluripotency using flow cytometry for the expression of the pluripotent markers (KYOUDXR0109B Human Induced Pluripotent Stem (IPS) Cells [201B7] (ATCC ACS1023)). The line of NCRM5-AAVS1-CAG-EGFP (clone 5), ND2-AAVS1-iCAG-tdTomato (clone 1), NCRM6, and MS19-ES-H were obtained from the NHLBI iPSC Core Facility of National Heart, Lung and Blood Institute (NHLBI). NCRM5-AAVS1-CAG-EGFP is an EGFP-expressing reporter iPSC line with CAG-EGFP targeted mono-allelically at the AAVS1 safe harbor locus in NCRM5 iPSCs. These iPSCs were reprogrammed from CD34^+^ peripheral blood mononuclear cells (PBMCs) from a healthy male donor and the cell line was authenticated (Luo et al., 2014). ND2-AAVS1-iCAG-tdTomato is a tdTomato-expressing reporter iPSC line with insulated CAG-tdTomato targeted mono-allelically at the AAVS1 safe harbor locus in ND2 iPSCs. These ND2-AAVS1-iCAG-tdTomato (clone1) cell line was reprogrammed using healthy male fibroblast cells and was authenticated by STR profiling performed by WiCell Cytogenetics lab using a Powerplex 16 System (Promega) and genomic DNA extracted from the iPSCs with DNeasy Blood and Tissue Kit (QIAGEN) (Patterson et al., 2020). Microglia differentiated from these two reporter lines were used for the xenotransplantation experiments. The MS19-ES-H line was reprogrammed from PBMCs from a healthy female donor with a Cytotune Sendai Virus kit (Thermo Fisher). The STR profiling was performed by WiCell Cytogenetics lab using a Powerplex 16 System (Promega) and genomic DNA extracted from the iPSCs with DNeasy Blood and Tissue Kit (QIAGEN) (Patterson et al., 2019). NCRM6 iPSC line was reprogrammed from CD34^+^ PBMCs from a healthy female donor with episomal iPSC reprogramming vectors (Thermo Fisher). This cell line was authenticated by Cell Line Genetics for STR service using the above same method. All hiPSC lines used in this study were determined by mycoplasma with MycoAlert (Lonza’s MycoAlert Plus kit) and excluded mycoplasma contamination.

Cells were cultured on Geltrex-coated (0.2 mg/ml, Gibco, #A1413302) 6-well plates using 1× mTeSRTM-1 medium. Passaging was performed following dissociation with TrypLE Express enzymatic digestion (Gibco by Life Technologies). Upon initial plating, cells were cultured in medium containing 3 µM Rho-kinase inhibitor (Y-27632, Abcam). The medium was completely refreshed every day. Cells reaching 70% confluence were either passaged or cryopreserved in Stem Cell Freezing Media (mFreSR, StemCells, Catalog # 05854).

### Myeloid progenitor cell differentiation and microglial cell maturation

The protocol for the differentiation of hiPSC cells to myeloid progenitors and then to microglia were adapted and modified from those described previously by the Cowley laboratory (van Wilgenburg et al., 2013; Haenseler et al., 2017). The first step of the protocol to enable EB formation employed the Spin-EBs formation method performed in AggreWellsTM800 microwell culture plates (Stemcell Technologies, Catalog # 34825). Briefly, 1 ml of mTeSRTM-1 spin-EB medium was added into each culture well and centrifuged at 3000 × *g* for 2 min. Subsequently, 4 × 10^6^ iPSCs in 1 ml of medium were added to the well of spin-EB plate and then centrifuged at 800 rpm for 3 min. The plate was incubated at 37°C, 5% CO_2_ for 4 days, then 1 ml medium was replaced in a drop-wise manner every day for the next 4 days with an EB medium: mTeSR1 medium (STEMCELL Technologies) containing 50 ng/ml BMP-4 (Gibco- PHC9534), 20 ng/ml human stem cell factor (SCF, Miltenyi Biotec), and 50 ng/ml vascular endothelial growth factor (hVEGF, Gibco- PHC9394).

In the second step of myeloid progenitor differentiation, the resulting EBs were harvested with a 40-µm filter column. Approximately 150–200 EBs were transferred into a 75-cm^2^ flask containing myeloid cell differentiation medium: TheraPEAK X-VIVO-15 Serum-free Hematopoietic Cell Medium (Lonza, Cat#: BEBP04-744Q) containing 100 ng/ml M-CSF (Invitrogen), 25 ng/ml IL-3 (R&D), 2 mM Glutamax supplement (Invitrogen), 1× N2 supplement (Thermo Fisher Scientific, Cat#17502048). Two-thirds of the media volume in the culture flask was replaced every 5 days for 2–3 weeks.

In the third step of microglia differentiation, the non-adherent floating cell layer consisting of differentiated myeloid progenitor cells was harvested from the supernatant and transferred into the 6-well plate and allowed to settle and adhere overnight. Two-thirds of the medium volume was removed and replaced with a microglia cell differentiation medium: DMEM/F12 medium (Gibco, #11330) containing 50 ng/ml M-CSF (Invitrogen), 100 ng/ml IL-34 (Peprotech), 10 ng/ml TGFb1 and 2–5 ng/ml TGFb2 (both from R&D Systems), 20 ng/ml CX3CL1 (Peprotech), 1× N2 supplement and 2 mM Glutamax supplement. The culture was maintained for 2 weeks after which the differentiated iPSC-derived microglia were harvested for analysis or for cell transplantation.

### Phagocytosis assay

To assess microglia phagocytosis in vitro, the following bioparticles were employed as targets: (1) pHrodo Red *E. coli* BioParticles Conjugate for Phagocytosis (Thermo Fisher Scientific, Cat#P35361); (2) pHrodo Red Zymosan Bioparticles Conjugate for Phagocytosis (Thermo Fisher Scientific, Cat#P35364); (3) bovine rod POSs (Invision Bioresources, Cat#98740). Bovine POS was diluted in serum-free DMEM/F12 (1:1; Gibco) to a concentration of 10^6^ segments/ml and fluorescently labeled with the lipophilic dye DiI (Vybrant Cell-Labeling Solutions; Invitrogen) according to the manufacturer’s instructions. Each phagocytosis assay employed 1 × 10^5^ POSs and 2 mg/ml pHrodo Red *E. coli* membrane/Zymosan BioParticles.

For the assay, harvested floating myeloid cells were transferred into 4-well chamber slides (Thermo Fisher). For phagocytosis assessment of myeloid cells, the cells were cultured for 1 day and challenged with bioparticles. To assess iPSC-derived microglia, the cells were cultured in a microglia cell differentiation medium for 2 weeks and then challenged. In the assay, bioparticles were added to the 100 µl serum-free DMEM/F12 medium in the slide chamber, incubated for 1 hr at 37°C, washed three times with phosphate-buffered saline (PBS), and then fixed in 4% paraformaldehyde (PFA) for 20 min. Fixed cells were immunostained with antibodies to IBA1 and P2RY12 and counterstained with DAPI. Stained cells were imaged with an Olympus 1000 confocal microscope, and image analysis was conducted using ImageJ software (NIH).

### mRNA and protein analysis following LPS challenge in vitro

Differentiated hiPSC-derived microglia cultured in 6-well plates were stimulated with LPS at 100 ng/ml for 6 and 24 hr. Microglia stimulated for 6 hr were collected in RNAlater solution (Thermo Fisher) and stored at −80°C for further qRT-PCR analysis. Microglia stimulated for 24 hr were collected for protein quantification. After the medium was collected, the cells in the well were washed with 1× PBS, and then 200 µl of RIPA lysis buffer with proteinase inhibitor cocktail (Calbiochem) was added; the cells were removed by scraping, collected into 1.5 ml Eppendorf tube, and then homogenized with sonication (Sonicator 125 Watts, Qsonica) at 4°C. After sonication and centrifugation, total protein concentration was measured (BCA protein assay kit; Pierce). Levels of individual cytokines were determined using a Milliplex bead assay kit (Milliplex MAP human cytokine/chemokine magnetic bead panel, #MCYTOMAG-70K; Millipore) and involving the Luminex MAPIX system with data analysis using xPONENT 4.2 software (Luminex). The cytokines analyzed included *IL1A*, *IL1B*, *IL6*, *IL8*, *TNFa*, *CXCL10*, *CCL2*, *CCL3*, *CCL4*, and *IL10*.

### mRNA expression analysis by quantitative RT-PCR

mRNA expression was quantitated using qRT-PCR. Harvested cells were lysed by trituration and homogenized using QIAshredder spin columns (QIAGEN). Total RNA was isolated using the RNeasy Mini kit (QIAGEN) according to the manufacturer’s specifications. First-strand cDNA synthesis from mRNA was performed using qScript cDNA SuperMix (Quanta Biosciences) using oligo-dT as primer. qRT-PCR was performed using an SYBR green RT-PCR kit (Affymetrix), using the Bio-Rad CFX96 Touch Real-Time PCR Detection System under the following conditions: denaturation at 95°C for 5 min, followed by 40 cycles of 95°C for 10 s, and then 60°C for 45 s. Threshold cycle (CT) values were calculated and expressed as fold-induction determined using the comparative CT (2ΔΔCT) method. Ribosomal protein S13 (RPS13) and GAPDH were used as internal controls. Oligonucleotide primers are provided in Supplementary file 4.

### Transplantation of hiPSC-derived microglia by subretinal injection

Differentiated hiPSC-derived microglia grown in flasks were first washed in 1× PBS before being removed by scraping and collected into a 50-ml tube in 5 ml PBS. Cell numbers were counted using a cell counter (Countess 3, Thermo Fisher). Microglia were collected by centrifugation (5 min at 4°C, 200 × *g*) and the resulting cell pellet resuspended in 1× PBS at a concentration of 5000 cells/µl for in vivo transplantation via subretinal injection. Experimental animals were given general anesthesia (ketamine 90 mg/kg and xylazine 8 mg/kg) and additional topical anesthesia (0.5% Proparacaine HCL, Sandoz) applied to the injected eye. For the injection, the temporal sclera was exposed by a conjunctival cut-down and a scleral incision made 0.5 mm behind the limbus using 32 G needle to access the subretinal space. The tip of a blunt 32 G needle attached to a Hamilton micro-syringe was introduced through the incision at an angle 5 degrees tangent to the globe and advanced 0.5–1 mm into the subretinal space under a dissecting microscope. Microglial cells (5000 cells in 1 µl PBS) were slowly injected from the micro-syringe into the subretinal space using an aseptic technique. Post-procedure, treated eyes were carefully inspected for signs of bleeding or distention and intraocular pressure was monitored using a tonometer (iCare TONOLAB, Finland). In the event that intraocular pressure remained elevated (>20 mmHg) and/or the globe appeared distended, a vitreous tap was performed using a 33 G needle to reduce intraocular pressure. In the unlikely event that excessive bleeding is observed, the animal will be examined by a veterinarian or euthanized immediately.

### Immunohistochemical analyses

For immunohistochemical analysis of microglia in vitro, microglia were differentiated in 4-well chambered slides, fixed in 4% PFA for 20 min and processed for immunostaining. For in vivo analyses, recipient animals were euthanized by CO_2_ inhalation, and the eyes were enucleated. Enucleated eyes were dissected to form posterior segment eyecups and fixed in 4% PFA in phosphate buffer (PB) for 2 hr at 4°C. Eyecups were either cryosectioned (Leica CM3050S) or further dissected to form retinal flat mounts. Flat-mounted retinas were blocked for 1 hr in a blocking buffer containing 10% normal donkey serum and 1% Triton X-100 in PBS at room temperature. Primary antibodies included IBA1 (1:500, Wako, #019-19741), anti-mouse Tmem119 (1:500, Synaptic Systems, #400 004), anti-human TMEM119 (1:100, Sigma, #HPA051870), anti-mouse Cd68 (1:200, Bio-Rad, #MCA1957), anti-human CD68 (1:100, R&D, #MAB20401), anti-mouse Cd45 (1:100, Bio-Rad, #MCA1388), anti-human CD45 (1:100, R&D, #FAB1430R), cone arrestin (1:200, Millipore, #AB15282), Ki67 (1:30, eBioscience, #50-5698-82), anti-P2RY12 (1:100, Thermo Fisher, #PA5-77671 and Sigma, #HPA014518), CD34 (1:50, eBioscience, #14–0341), hCD11b (1:100, R&D, #FAB1699R), mCD11b (Bio-Rad, Cat#: MCA711G, 1:100), CX3CR1 (1:100, Invitrogen, #61-6099-42), hHLA (Invitrogen, #11-9983-42), SPI1 (Invitrogen, #MA5-15064), TREM2 (1:100, Invitrogen, #702886), glutamine synthetase (1:200, Millipore, #MAB302), PKCa (1:200, Sigma-Aldrich, #p4334), GFAP (1:200, Invitrogen, #13-0300), RBPMS (1:100, Phosphosolutions, 1832-RBPMS), Calbindin (1:5000, Swant, CB-38a), and anti-RFP (1:200, RockLand, 600-401-379-RTU). Primary antibodies were diluted in blocking buffer and incubated at 4°C overnight for retinal sections and at room temperature overnight on a shaker for retinal flat mounts. Experiments in which primary antibodies were omitted served as negative controls. After washing in 1× PBST (0.2% Tween-20 in PBS), retinal samples were incubated for 2 hr at room temperature with secondary antibodies (AlexaFluor 488-, 568-, or 647-conjugated anti-rabbit, mouse, rat, goat, and guinea pig IgG) and DAPI (1:500; Sigma-Aldrich) to label cell nuclei. Isolectin B4 (IB4), conjugated to AlexaFluor 568/647 (1:100, Life Technologies), was used to label activated microglia and retinal vessels. Stained retinal samples were imaged with confocal microscopy (Olympus FluoView 1000, or Zeiss LSM 880, or Nikon A1R). For analysis at high magnification, multiplane z-series were collected using 20 or 40 objectives; Confocal image stacks were viewed and analyzed with FV100 Viewer Software, NIS-Element Analysis and ImageJ (NIH).

### RNAseq analysis

For whole transcriptome analysis, cultured myeloid progenitor cells and differentiated microglial cells with and without 0.1 µg/ml LPS treatment were harvested in the flasks and 6-well plates, respectively. After harvesting, all samples stored frozen in RNAlater (Roche) solution before RNA extraction using the QIAGEN RNA Mini Kit. RNA quality and quantity were evaluated using Bioanalyzer 2100 with the RNA 6000 Nano Kit (Agilent Technologies). The preparation of RNA library and transcriptome sequencing was performed using an external vendor (Novogene, Sacramento, CA). Genes with adjusted p-value <0.05 and log2FC (Fold Change) >1 were considered differentially expressed. IPA (QIAGEN) was employed for canonical pathway and graphical pathway analysis. The microglia gene list was constructed from our previous microarray data from retinal microglial cells and published data (Ma et al., 2013; Bennett et al., 2016). The heat map, volcano, and histogram plot were performed using Prism 9.5.1 (GraphPad).

### NaIO_3_-induced model of RPE cell injury

Recipient mice 8 months following xenotransplantation were administered a single dose of NaIO_3_ (Honeywell Research Chemicals) at a dose of 30 mg/kg body weight via intraperitoneal injection. Animals were euthanized 3 and 7 days after NaIO_3_ injection, and their retinas were harvested and subjected to histological and molecular analysis.

### Statistics and reproducibility

All data in the graphical panel represent mean ± standard error. When only two independent groups were compared, signiﬁcance was determined by a two-tailed unpaired *t*-test with Welch’s correction. When three or more groups were compared, one-way ANOVA with the Bonferroni post hoc test or two-way ANOVA was used. A p-value <0.05 was considered signiﬁcant. The analyses were done in GraphPad Prism v.5. All experiments were independently performed at least three experimental replicates to confirm consistency in observations across replicates.

## Data Availability

All data generated or analyzed during this study are included in the manuscript and supporting files; source data files have been provided for Figures 2 and 3 and Figure 2 suppl. A, B, and C. The following previously published datasets were used: AbudEM RamirezRN MartinezES HealyLM NguyenCHH NewmanSA YerominAV ScarfoneVM MarshSE FimbresC CarawayCA FoteGM MadanyAM AgrawalA KayedR GylysKH CahalanMD CummingsBJ AntelJP MortazaviA CarsonMJ PoonWW Blurton-JonesM 2017Generation of human microglia-like cells to study neurological diseaseNCBI Gene Expression OmnibusGSE8918910.1016/j.neuron.2017.03.042PMC548241928426964 an der PoelM UlasT MizeeMR HsiaoCC MiedemaSSM AdeliaSKG HelderB TasSW SchultzeJL HamannJ HuitingaI 2019Transcriptional profiling of human microglia reveals grey-white matter heterogeneity and multiple sclerosis-associated changesNCBI Gene Expression OmnibusGSE11197210.1038/s41467-019-08976-7PMC641631830867424

## Additional files

Supplementary file 1. Microglia-enriched gene list. The gene list of 71 microglia-enriched genes was extracted from bulk RNAseq of microglial cells vs. myeloid progenitor cells (MPCs). The total microglia-enriched gene list combined from the research conducted by Barres BA Lab (Bennett et al., Proc Natl Acad Sci U S A, 2016) and from our RNA sequencing data of mouse retinal microglia (Ma et al., 2013), identifying a total of 130 genes predominantly expressed in microglia.

Supplementary file 2. 188 hMG gene comparisons vs. GSE178846. The human microglia gene panel combined our mouse microglia-enriched genes and human microglia-enriched genes (Abud et al., 2017; Muffat et al., 2016; Douvaras et al., 2017; Böttcher et al., 2019; van der Poel et al., 2019). The total microglia-enriched gene list contains 203 genes, which used to be extracted from the gene profile of hiPSC-MG and downloaded human adult microglia (AMG), fetal microglia (FMG), inflammatory monocyte (IM) and monocytes (M) (GSE 178846, Abud et al., 2017). 188 genes were obtained from both gene lists. All the gene counts were normalized with four human cells housekeeping genes C1orf43, RAB7A, REEP5, and VCP (Eisenberg and Levanon, 2013).

Supplementary file 3. The gene list of comparison vs. GSE111972. The total gene list of both male and female hiPSC-derived microglial cells and human brain microglia cells (GSE 111972, van der Poel et al., 2019).

Supplementary file 4. The oligos for quantitative reverse transcription-PCR (qRT-PCR). The oligos were used for qRT-PCR in cultured hiPSC-derived cells.

Supplementary file 5. Human-specific oligos for quantitative reverse transcription-PCR (qRT-PCR). The oligos were used for qRT-PCR in mouse retinas with integrated hiPSC-derived microglial cells.

Supplementary file 6. 42 genes associated with age-related macular degeneration (AMD). 42 candidate genes from 34 loci associated with AMD expressed in retinal microglia cells. The microglia gene expression data are from microarray data previously published (Ma et al., 2013). The candidate genes came from the published paper (den Hollander et al., 2022).

Supplementary file 7. 209 genes associated with age-related macular degeneration (AMD). The 209 microglia-enriched genes were extracted from the genes associated with AMD (Fritsche et al., 2016). The microglia gene expression data are from microarray data previously published (Ma et al., 2013).

MDAR checklist
